# Artificial Olfactory System Enabled by Ultralow Chemical Sensing Variations of 1D SnO_2_ Nanoarchitectures

**DOI:** 10.1002/advs.202501293

**Published:** 2025-05-03

**Authors:** Yun‐Haeng Cho, Dong‐Su Kim, Jung Hwan Seo, Jae Han Chung, Zion Park, Ki Chang Kwon, Jae‐Kwon Ko, Tae Won Ha, Jeong‐O Lee, Gyu‐Li Kim, Seong‐Jun Ro, Hyojung Kim, Chil‐Hyoung Lee, Kwangjae Lee, Young‐Seok Shim, Donghwi Cho

**Affiliations:** ^1^ School of Energy Materials and Chemical Engineering Korea University of Technology and Education (KOREATECH) Cheonan 31253 Republic of Korea; ^2^ National Center for Nano Process & Equipments, Energy & Nano Technology Group Korea Institute of Industrial Technology (KITECH) Gwangju 61012 Republic of Korea; ^3^ Department of Mechanical Engineering Hongik University Seoul 04066 Republic of Korea; ^4^ Division of Chemical and Material Metrology Korea Research Institute of Standards and Science (KRISS) Daejeon 34113 Republic of Korea; ^5^ Department of Analytical Science and Technology Graduate School of Analytical Science and Technology (GRAST) Chungnam National University Daejeon 34134 Republic of Korea; ^6^ Thin Film Materials Research Center Korea Research Institute of Chemical Technology (KRICT) Daejeon 34114 Republic of Korea; ^7^ Department of AI Mobility Engineering Sangmyung University Cheonan 31066 Republic of Korea; ^8^ Department of Semiconductor Systems Engineering Sejong University Seoul 05006 Republic of Korea; ^9^ Advanced Materials and Chemical Engineering University of Science and Technology Daejeon 34113 Republic of Korea

**Keywords:** artificial olfactory system, deep learning, gas sensor, glancing angle deposition, nanoarchitectures, SnO_2_

## Abstract

AI‐assisted electronic nose systems often emphasize sensitivity‐driven datasets, overlooking the comprehensive analysis of gaseous chemical attributes critical for precise gas identification. Conventional fabrication methods generate inconsistent datasets and focus primarily on improving classification accuracy through deep learning, neglecting the fundamental role of sensor material design. This study addresses these challenges by developing a highly reliable sensor platform to standardize gas sensing for deep learning applications. Specifically, 1D SnO_2_ nanonetworks functionalized with Au and Pd nanocatalysts are fabricated via a systematic deposition process, enhancing gas diffusion and reaction kinetics. Stability improvements through controlled aging process reduce the coefficient of variation to below 5% across seven target gases: acetone, hydrogen, ethanol, carbon monoxide, propane, isoprene, and toluene. The platform exhibits exceptional deep learning performance, achieving over 99.5% classification accuracy using a residual network model, even in high‐humidity environments (up to 80% relative humidity) and at parts‐per‐trillion detection limits. This study highlights the synergy between nanostructure engineering and AI, establishing a robust framework for next‐generation bioinspired electronic nose systems with enhanced reliability and analytical capability.

## Graphical abstract

This study presents a highly reliable gas sensor platform featuring SnO_2_ nanonetworks functionalized with Au and Pd nanocatalysts. Enhanced stability and optimized performance enable over 99.5% classification accuracy in deep learning, even under extreme conditions. This work highlights the synergy of nanostructure design and AI, advancing bioinspired electronic nose systems.

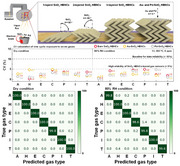

## 1 Introduction

Over the past few decades, metal oxide semiconductor (SMO) gas sensors have emerged as promising candidates for next‐generation gas‐sensing technologies due to their scalability, cost efficiency, and versatility in integration.^[^ ^1^, ^2^ ^]^ Advances in nanostructure design and catalyst incorporation have significantly enhanced the sensitivity and selectivity of these sensors, enabling detection of trace concentrations of target gases.^[^ ^3^, ^4^, ^5^ ^]^ However, challenges such as limited selectivity and cross‐sensitivity continue to impede the development of electronic noses that can replicate or surpass the capabilities of the human olfactory system.^[^ ^6^, ^7^, ^8^, ^9^, ^10^ ^]^

The advent of deep learning has revolutionized multiple fields, offering opportunities to emulate and enhance human sensory capabilities in gas sensing.^[^ ^11^ ^]^ By analyzing complex data patterns that are influenced by variables such as temperature and humidity, deep learning algorithms enable precise identification of target gases under challenging conditions.^[^ ^12^ ^]^ SMO sensors, in particular, have greatly benefited from this synergy, where nanomaterial‐based sensor arrays combined with AI‐driven algorithms support the creation of highly sensitive and selective detection systems.^[^ ^13^, ^14^ ^]^ However, most studies primarily focus on data processing, often overlooking critical factors such as material reliability, dataset reproducibility, and the impact of sensing material morphology on sensor performance. These gaps highlight the need for rigorous material optimization as a foundational step toward achieving high accuracy and reliability in deep learning‐based gas‐sensing systems.

Reliable fabrication techniques, such as e‐beam deposition, sputtering, and thermal evaporation, are essential for producing consistent sensing materials and minimizing the coefficient of variation (CV)—a key metric for assessing data reliability.^[^ ^15^, ^16^, ^17^ ^]^ While these methods enhance sensitivity and selectivity, the lack of systematic aging procedures restricts long‐term stability. Aging improves surface dynamics, such as adsorption–desorption equilibria, ensuring reproducible sensor behavior and generating high‐quality datasets for deep learning.^[^ ^18^ ^]^ A low CV is critical for meaningful data augmentation, which mimics real‐world variability and mitigates challenges such as high computational demands and reduced classification accuracy.^[^ ^19^ ^]^ Despite its significance, to be best of our knowledge, no studies have systematically addressed the minimization of CV while designing stable, reproducible gas sensors with comprehensive aging processes to support deep learning applications.

In this study, we propose a design strategy for an exceptionally uniform gas‐sensing platform based on 1D nanonetworks of highly periodic SnO_2_ herringbone‐like nanocolumns (HBNCs) functionalized with Au and Pd nanocatalysts. This platform achieves an ultralow CV below 5% and generates high‐quality datasets optimized for deep learning applications. First, an e‐beam deposition process was developed to optimize gas diffusion and chemical kinetics, with the incorporation of Au and Pd catalysts to enhance selectivity for specific target gases. Second, a systematic aging process was implemented to ensure stability and reproducibility, achieving CVs below 5% across seven target gases (acetone, hydrogen, ethanol, carbon monoxide, propane, isoprene, and toluene) under varying concentrations and humidity conditions. Third, we applied data augmentation using spectrogram‐based augmentation (SpecAugment) along with dynamic time warping (DTW)‐based upsampling, employed a Residual Network model for classification, and achieved accuracy exceeding 99.5%.^[^ ^20^, ^21^ ^]^ Robustness was validated through k‐fold cross‐validation, confirming the reliability of the proposed approach. This study establishes a comprehensive framework for designing high‐performance gas sensors, paving the way for significant advancements in electronic nose technologies.

## 2 Results and Discussion

### 2.1 Design of Highly Uniform SnO_2_ HBNCs for Reliable Sensing Properties

SMO gas sensors require sensing materials that exhibit structural, physical, and chemical stability, along with high responsiveness, to ensure accurate performance in deep learning‐based analyses. The glancing angle deposition (GLAD) method, utilizing an e‐beam evaporator, enables precise control over nanostructures such as vertical nanorods, inclined nanocolumns, and HBNCs by adjusting substrate rotation, deposition angle, and temperature.^[^ ^22^, ^23^ ^]^ This technique also facilitates tunable porosity, density, and diameter through stacked layer configurations, enhancing its applicability in gas sensing.^[^ ^24^ ^]^ Notably, the design of nanocolumns with a thickness approximately twice the Debye length (2𝜆𝐷) improves surface charge modulation and enhances gas‐sensing properties.^[^ ^25^, ^26^ ^]^ As a case study for AI‐assisted gas‐sensing applications, SnO_2_ HBNCs were fabricated using the GLAD method.

**Figure 1:**
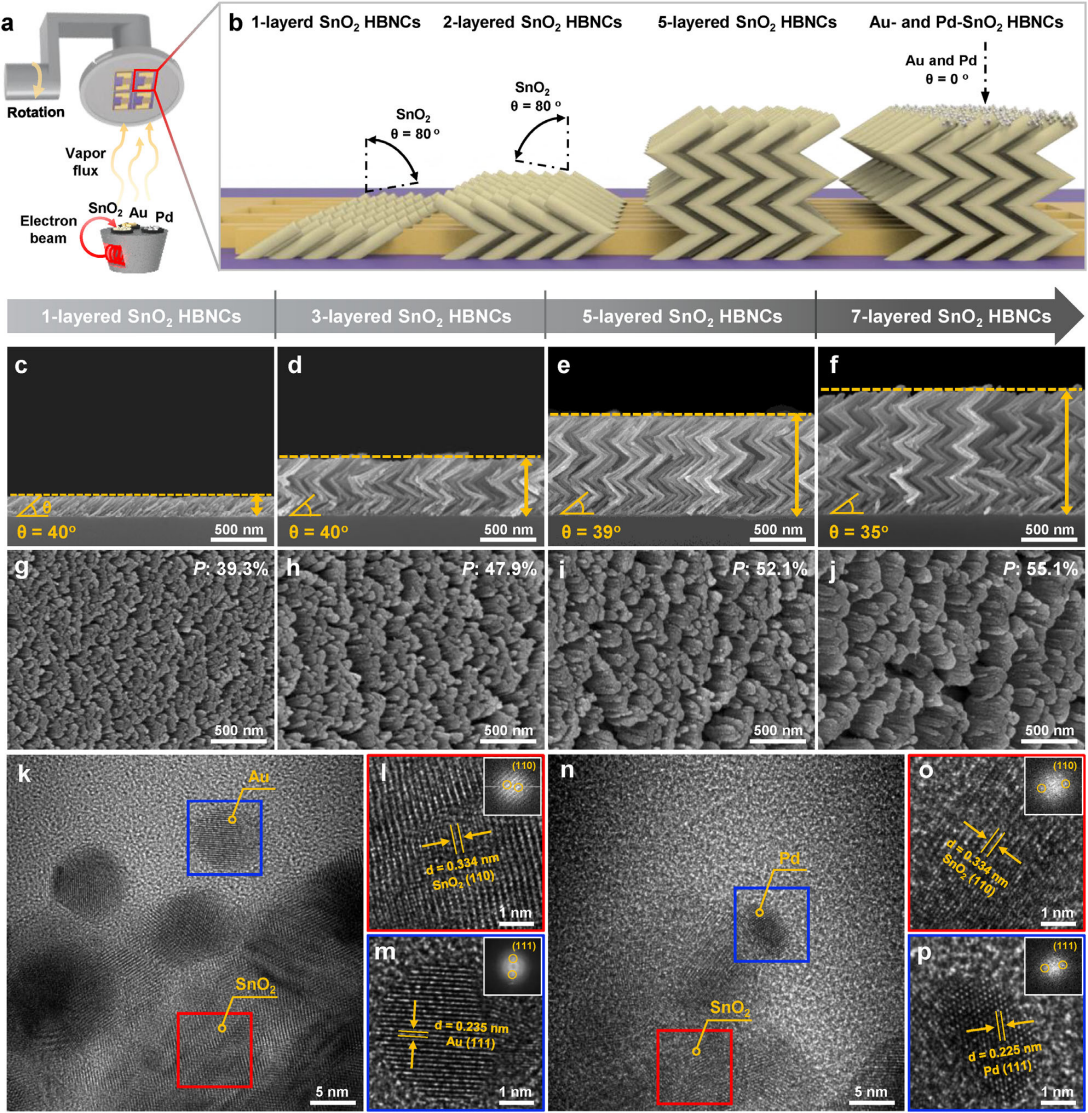
Design concept of SnO_2_ HBNCs. a) Schematic illustration of the deposition process for SnO_2_ HBNCs with Au and Pd catalysts using e‐beam evaporation. b) Structural evolution as a function of the deposition angle. c–f) Cross‐sectional and g–j) top‐view SEM images of SnO_2_ HBNCs with varying numbers of layers: 1‐, 3‐, 5‐, and 7‐layered SnO_2_ HBNCs. k) HR‐TEM image of Au‐SnO_2_ HBNCs and FFT pattern of l) SnO_2_ and m) Au NP. n) HR‐TEM image of Pd‐SnO_2_ HBNCs and FFT pattern of o) SnO_2_ and p) Pd NP.

**Figure** **1**a,b illustrates the systematic GLAD procedures employed to fabricate SnO_2_ HBNCs, decorated with Au and Pd. Substrate alignment ensured that the long fingers of the interdigitated electrodes (IDEs) were parallel to the deposition direction of the inclined nanocolumns, promoting the formation of HBNCs between electrodes while minimizing shadowing effects from IDE sidewalls (Figure S1, Supporting Information). A sequential deposition process was employed to control the number of stacked layers, with each layer forming ≈180 nm‐thick SnO_2_ inclined nanocolumns, referred to as “1‐layered SnO_2_ HBNCs,” as described in the Experimental Section.

Scanning electron microscopy (SEM) analysis revealed the ordered morphologies of SnO_2_ HBNCs as the number of stacked layers increased (Figure 1c–f). Despite maintaining a fixed deposition rate, structural shrinkage was observed due to the weight of the upper nanocolumns, resulting in thicknesses of 180, 540, 890, and 1150 nm for the 1‐, 3‐, 5‐, and 7‐layered SnO_2_ HBNCs, respectively. The first layer was deposited directly onto the SiO_2_/Si substrate, while subsequent layers nucleated on the pre‐formed nanocolumns, increasing shadowing effects and reducing the total thickness per layer. The angle of the initial layer decreased from 40° to 36° in the 7‐layered SnO_2_ HBNCs, leading to changes in porosity and nanocolumn diameter (Figure 1g–j). MATLAB analysis of tilted‐view SEM binary images (Figure S2, Supporting Information) quantified porosity ratios based on SnO_2_ (white) and void (black) areas, revealing an increase in porosity with each additional layer (39.3%, 47.9%, 52.1%, and 55.1%, respectively).

To investigate the morphological changes and distribution of Au and Pd catalysts based on initial thickness, Au and Pd films (0.5, 1, 3, 5, and 10 nm) were deposited on SiO_2_/Si substrates and analyzed after thermal treatment (Figure S3, Supporting Information). Increasing the Au film thickness led to self‐agglomeration and larger nanoparticles (NPs). In contrast, Pd films exhibited limited agglomeration, forming smaller NPs and gradually covering the SiO_2_/Si substrate. This difference arises from lower surface diffusivity and higher melting point of Pd compared to Au, requiring greater thermal activation energy for NP formation.^[^ ^27^, ^28^ ^]^

To optimize catalyst distribution, Au and Pd films of varying thicknesses were deposited on SnO_2_ HBNCs and annealed. SEM images reveal that increasing the Au film thickness from 0.5 to 10 nm resulted in Au NPs growing from ≈9 to 269 nm (Figure S4a,b, Supporting Information). Notably, at 10 nm, Au did not form distinct NPs but instead created interconnections between nanocolumns. For Pd, increasing thickness led to gradual thickening and dense surface coverage, leading to the formation of conductive pathways and a reduction in neck contacts (Figure S4c,d, Supporting Information). However, Pd NPs were not observed on SnO_2_ HBNCs. In SEM analysis, atomic number differences influence contrast due to enhanced backscattered electron signals. The atomic number difference between Pd and Si (46 vs 14) is significant, improving visibility. However, Pd and Sn (46 vs 50) are closer in atomic number, making differentiation more challenging.

To investigate the crystallinity and the formation of Au and Pd NPs on SnO_2_ HBNCs, 1 nm‐Au‐ and Pd‐decorated SnO_2_ HBNCs (referred to as “Au‐SnO_2_ HBNCs” and “Pd‐SnO_2_ HBNCs”) were characterized by high‐resolution transmission electron microscopy (HR‐TEM) analysis. The rutile phase of SnO_2_, with a fringe spacing of 0.334 nm corresponding to the (110) d‐spacing (JCPDS #770 449) was confirmed in Figure 1k–p and X‐ray diffraction (XRD) analysis further supported these findings (Figure S5, Supporting Information). The formation of Au and Pd NPs was observed in the blue boxes in Figure 1k,n and did not significantly alter the morphology of SnO_2_ HBNCs, as well as in Figure S6 (Supporting Information). Fast Fourier transform (FFT) pattern analysis revealed fringe spacings of 0.235 and 0.225 nm for Au and Pd NPs, respectively, which correspond to a preferred (111) orientation that minimizes surface energy during annealing (Figure 1m,p).^[^ ^29^ ^]^

X‐ray photoelectron spectroscopy (XPS) analysis was conducted to investigate the chemical interactions between SnO_2_ and the NPs (Figure S7, Supporting Information). The Sn 3d and O 1s peaks of Au‐ and Pd‐SnO_2_ HBNCs shifted to lower binding energies compared to bare SnO_2_ HBNCs, indicating charge migration from the SnO_2_ layer to the NPs. Notably, Pd‐SnO_2_ HBNCs exhibited more pronounced binding energy shifts than Au‐SnO_2_ HBNCs, suggesting a stronger electron transfer in the Pd‐SnO_2_ system.

### 2.2 Systematic Optimization of Morphology and Catalyst Distribution in SnO_2_ HBNCs

The gas sensing performance of SnO_2_ HBNCs is strongly influenced by structural characteristics, including porosity, density, and thickness. Multi‐stacked SnO_2_ HBNCs exhibit higher porosity and thicker nanocolumns compared to single‐layered nanocolumns. An adequate balance between porosity and nanocolumn diameter is critical for enhancing gas diffusion and surface chemical reactions. Small pores (<2 nm) offer a large surface area but provide insufficient pathways for gas diffusion within the sensing layer.^[^ ^30^, ^31^ ^]^ In contrast, large pores (>50 nm) facilitate gas diffusion but allow gas molecules to freely pass without sufficient adsorption on the surface, and they have a relatively lower surface area compared to sensing materials with smaller pores. Meanwhile, thicker nanocolumns hinder the formation of sufficient depletion layers, which are critical for chemiresistive changes and improved responses to target gases. Additionally, thicker structures reduce the number of neck contacts between nanocolumns compared to one‐layered SnO_2_ HBNCs, limiting variations in the Schottky barrier height and thereby diminishing resistance changes in the conduction channel. Therefore, optimizing the form factors of SnO_2_ HBNCs is essential to maximize gas sensing performance, including response, response time, and recovery time. To evaluate these effects, samples with different deposition angles (0°, 75°, 80°, and 85°) were fabricated to have similar thicknesses, and all sensors underwent a 2‐week aging process under ambient conditions to induce crystal size changes and sensitivity degradation, ensuring long‐term stability and reliable performance (Figures S8 and S9, Supporting Information).^[^ ^32^ ^]^

**Figure 2:**
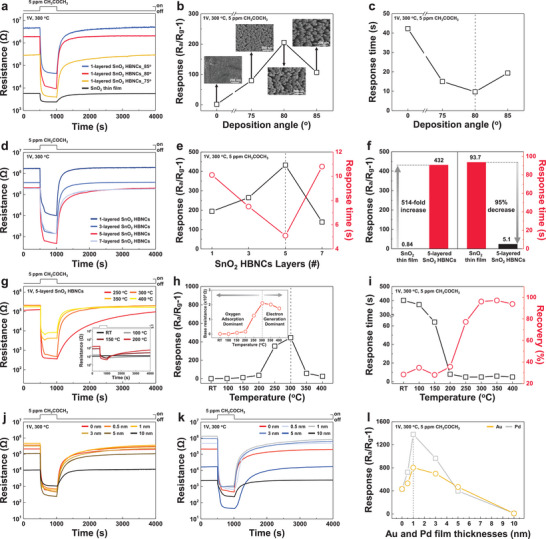
Optimization of the structural and physicochemical properties of SnO_2_ HBNCs. a) Response curves and b) responses, c) response times and recovery times to 5 ppm CH_3_COCH_3_ at 300 °C for SnO_2_ thin film and one‐layered SnO_2_ HBNCs as a function of deposition angle: 75°, 80°, and 85°. The insets in (b) show top‐view SEM images. d) Response curves and e) responses and response times to 5 ppm CH_3_COCH_3_ at 300 °C for SnO_2_ HBNCs with varying numbers of layers: 1‐, 3‐, 5‐, and 7‐layered SnO_2_ HBNCs. f) Comparison of response and response time between SnO_2_ thin film and 5‐layered SnO_2_ HBNCs. g) Response curves, h) responses, and i) response times and recovery of the intrinsic resistance of 5‐layered SnO_2_ HBNCs as a function of temperature. The inset in (h) shows the baseline resistance of 5‐layered SnO_2_ HBNCs at varying operating temperatures. Response curves of j) Au‐ and k) Pd‐decorated SnO_2_ HBNCs and l) responses to 5 ppm CH_3_COCH_3_ at 300 °C as a function of the initial thickness of Au and Pd films: 0, 0.5, 1, 3, 5, and 10 nm.

**Figure** **2**a presents resistance curves for the sensors upon exposure to 5 ppm CH_3_COCH_3_ at 300 °C, exhibiting typical n‐type semiconducting gas responses with full recovery to baseline resistance after gas removal. Among the samples, the 1‐layered SnO_2_ HBNCs deposited at 80° exhibited superior performance with the highest response (defined as (*R*_a_ − *R*_g_)/*R*_g_, where *R*_a_ and *R*_g_ represent the resistance in air and the target gas, respectively) of 204 and the fastest response time (*t*_90%_[air‐to‐gas]) of 9.7 s (Figure 2b,c). This enhanced performance is attributed to their structural characteristics, which supports effective gas diffusion and the formation of extensive depletion layers (Inset in Figure 2b).

To further investigate the morphological effects, samples with varying thicknesses (1, 3, 5, and 7 layers) were fabricated at optimal deposition angle and measured to 5 ppm CH_3_COCH_3_ at 300 °C (Figure 2d). The 5‐layered SnO_2_ HBNCs achieved the highest response of 432 and the fastest response of 5.1 s, which can be attributed to gas diffusion efficiency with adequate porosity and the formation of sufficient depletion layer (Figure 2e). However, the 7‐layered SnO_2_ HBNCs exhibited reduced response and slower response time, likely due to hindered gas diffusion and structural instability from excessive upper layers. Notably, the 5‐layered SnO_2_ HBNCs demonstrated a 514‐fold improvement in response (0.84 vs 432) and a 95% reduction in response time (93.7 s vs 5.1 s) compared to SnO_2_ thin film of similar thickness (890 nm) (Figure 2f and Figure S10, Supporting Information), underscoring their superior gas diffusion efficiency and electronic sensitization effects. A comparative analysis of SnO_2_ film with varying thicknesses (220, 470, 890, and 1520 nm) revealed that the base resistance gradually decreases due to the presence of additional conduction pathways in the film. Meanwhile, the gas response also decreases because of the limited surface area available to interact with gas molecules in the deeper sites.^[^ ^33^ ^]^

Optimization of the operating temperature is crucial for effective surface interactions, as the dominant ionized oxygen species (O^2−^, O^−^, O_2_^−^) vary with temperature.^[^ ^34^ ^]^ The 5‐layered SnO_2_ HBNCs were tested across temperatures ranging from 25 to 400 °C with 5 ppm CH_3_COCH_3_ (Figure 2g). As the temperature increased to 300 °C, the base resistance rose due to the transition of adsorbed oxygen species from O^2−^ to O^−^ but decreased above 300 °C due to excessive electron generation (Inset in Figure 2h). Gas desorption at higher temperatures created a volcano‐shaped response curve, with a peak response at 300 °C.^[^ ^35^ ^]^ Figure 2i shows the response time and the recovery percentage relative to the baseline resistance, after 3000 s of exposure to dry air, revealing rapid responses (<8 s) above 200 °C and complete recovery (*t*_10%_) above 300 °C, confirming 300 °C as the optimal operating temperature.

To evaluate gas sensing performance based on Au and Pd NP distribution, all samples were exposed to 5 ppm CH_3_COCH_3_ at 300 °C (Figure 2j,k). A volcano‐shaped response curve was observed, with the highest response at an initial Au and Pd film thickness of 1 nm on SnO_2_ HBNCs (Figure 2l). For example, at 0.5 nm thickness, Au and Pd NPs increased the electron depletion region but were too sparsely distributed to provide effective catalytic effects, leading to a lower response than at 1 nm (Figure S11a, Supporting Information). In contrast, the 1 nm‐thick Au and Pd films formed an optimal NP distribution, maximizing gas reactions and electronic sensitization (Figure S11b, Supporting Information). For thicker films (3–5 nm), Au and Pd NPs grew larger and became more sparsely distributed, reducing catalytic effects by limiting interfacial electron depletion region overlap, resulting in a weaker response than at 1 nm (Figure S11c, Supporting Information). At 10 nm, Au and Pd formed an overlayer on the surface, decreasing the active area, conductive pathways, and neck contacts. This led to lower base resistance and a diminished gas response compared to bare SnO_2_ HBNCs (Figure S11d, Supporting Information). Thus, the optimal Au and Pd NP distribution was achieved with 1 nm‐thick Au and Pd films, yielding the highest gas response.

The higher base resistance of Pd‐SnO_2_ HBNCs compared to Au‐SnO_2_ HBNCs indicates stronger electronic sensitization effects between SnO_2_ and Pd NPs, consistent with XPS results (Figure S7, Supporting Information). Both Au‐ and Pd‐SnO_2_ HBNCs demonstrated enhanced resistance transients and response values compared to bare SnO_2_ HBNCs. Notably, Au‐SnO_2_ HBNCs exhibited faster response time (reducing from 5.1 to 3.1 s), while Pd‐SnO_2_ HBNCs exhibited greater response enhancement (Figure S12, Supporting Information). These findings suggest that Au NPs contribute to both chemical and electronic sensitization, whereas Pd NPs primarily enhance electronic sensitization during gas reactions with CH_3_COCH_3_.

### 2.3 Exceptional Sensing Reliability of SnO_2_ HBNCs

**Figure 3:**
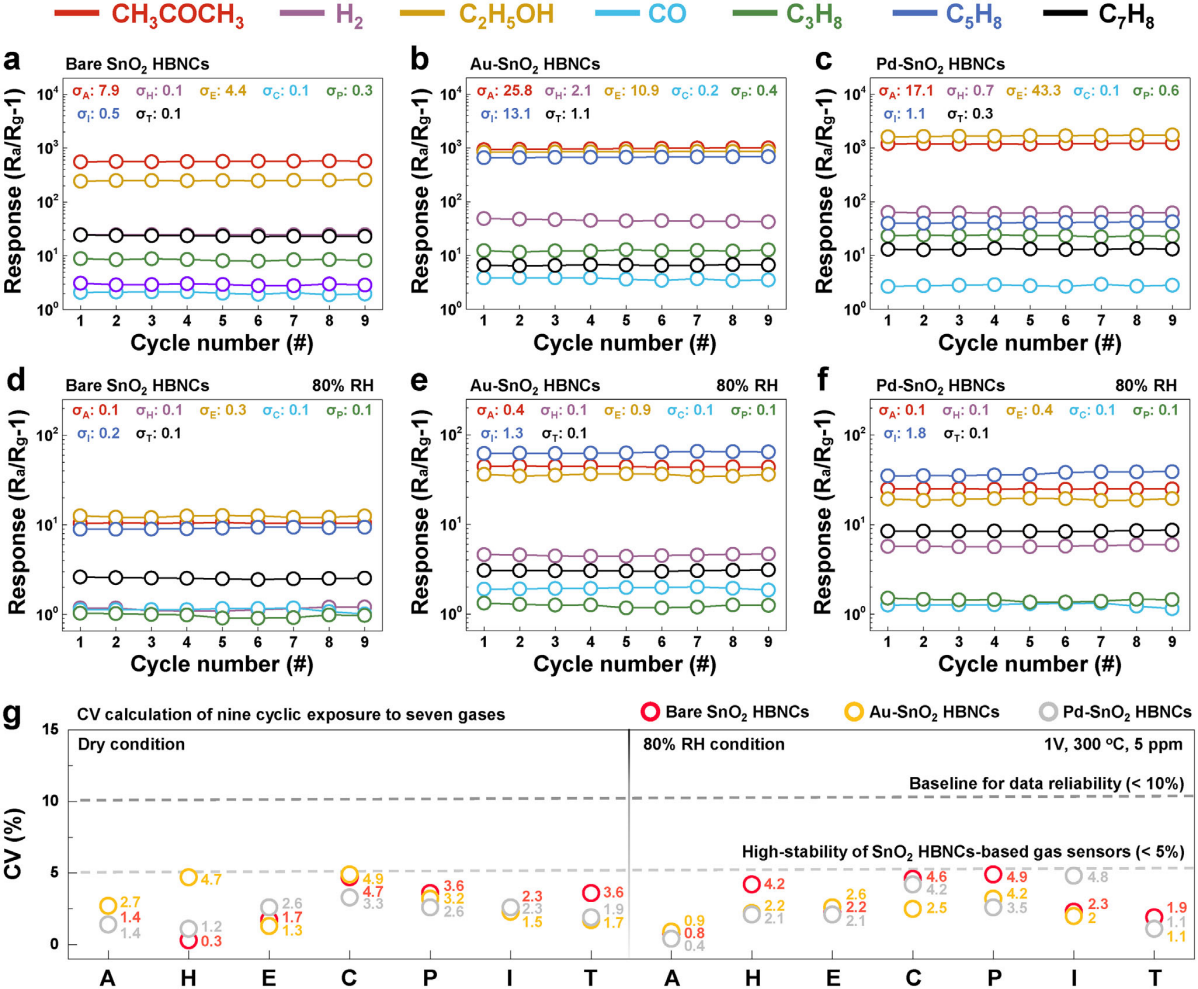
Sensing performances of SnO_2_ HBNCs‐based gas sensors. Summary of nine cyclic responses to seven gases at 5 ppm and 300 °C for a) bare, b) Au‐, and c) Pd‐SnO_2_ HBNCs under dry conditions, and d) bare, e) Au‐, and f) Pd‐SnO_2_ HBNCs under 80% RH conditions. g) Summary of CV values for seven gases measured using bare, Au‐, and Pd‐SnO_2_ HBNCs under both dry and 80% RH conditions (A: acetone, H: hydrogen, E: ethanol, C: carbon monoxide, P: propane, I: isoprene, and T: toluene).

The fabricated sensors, optimized with 5‐layered SnO_2_ HBNCs, a 1 V applied voltage, and a 300 °C operating temperature, were evaluated for stability, reliability, and performance variations using seven target gases (CH_3_COCH_3_, H_2_, C_2_H_5_OH, CO, C_3_H_8_, C_5_H_8_, and C_7_H_8_) (**Figure** **3**). These gases were selected for their relevance in industrial safety, environmental monitoring, and medical diagnostics. To assess detection capability, bare, Au‐, and Pd‐SnO_2_ HBNCs underwent nine repeated cyclic exposures to each gas at a fixed 5 ppm concentration. The results (Figure 3a–c) and corresponding resistance curves (Figure S13, Supporting Information) confirm reliable, sensitive cyclic response and recovery across all sensors. Notably, gases with high sensitivity (CH_3_COCH_3_, C_2_H_5_OH, and C_5_H_8_) exhibited shark tail‐shaped response curves, while low sensitivity gases (H_2_, CO, C_3_H_8_, and C_7_H_8_) initially showed an overshoot before stabilizing—a trend widely reported in previous studies.^[^ ^15^, ^36^, ^37^ ^]^ The overshoot effect in low sensitivity gases arises from weak chemical interactions with pre‐adsorbed oxygen species, leading to rapid physisorption followed by slower charge transfer and desorption, delaying equilibrium.^[^ ^38^, ^39^, ^40^ ^]^ To mitigate this effect, adjusting gas measurement conditions—such as reducing concentration or using temperature modulation—can regulate adsorption–desorption kinetics and charge redistribution, ensuring faster stabilization and minimizing transient response.^[^ ^41^, ^42^ ^]^

As depicted in Figure 3a, bare SnO_2_ HBNCs showed a response of ≈251 to 5 ppm C_2_H_5_OH. The response significantly increased upon incorporating Au and Pd NPs, reaching 849 for Au‐SnO_2_ HBNCs and 1677 for Pd‐SnO_2_ HBNCs (Figure 3b,c). The variations in response are attributed to the catalytic activity and gas adsorption properties of the Au and Pd NPs decorating the SnO_2_ HBNCs. During cyclic testing, all sensors demonstrated high stability and reliability, due to the aging process and structural uniformity, which facilitated effective gas diffusion through the channels. For CO detection, Au‐SnO_2_ HBNCs exhibited the highest response among the samples, with a value near 4, which can be attributed to the enhanced chemisorption of oxygen ions induced by Au NPs, facilitating efficient CO oxidation on the sensing materials.^[^ ^43^ ^]^ Furthermore, owing to the higher catalytic activity of Au for unsaturated hydrocarbons, Au‐SnO_2_ HBNCs efficiently detect C_5_H_8_ by promoting its conversion into acetaldehyde, thereby accelerating and enhancing the sensing performance.^[^ ^44^ ^]^

Similar sensing trends were observed for H_2_, C_2_H_5_OH, C_3_H_8_, and C_7_H_8_, highlighting the high catalytic efficiency of Pd. The enhanced performance of Pd‐SnO_2_ HBNCs can be attributed to several factors. Pd exhibits strong hydrogen adsorption properties, making it particularly effective for sensing H₂ and other reducing gases by facilitating the dissociation of H₂ into atomic hydrogen, thereby promoting charge transfer on the sensor surface.^[^ ^45^ ^]^ Additionally, the lower activation energy of Pd for catalytic oxidation enables more efficient oxidation of volatile organic compounds, resulting in higher responses. The strong chemical interactions between Pd and gas molecules further enhance adsorption and desorption kinetics, reducing response time. Together, these characteristics significantly improve the gas sensing performance of Pd‐SnO_2_ HBNCs.

Maintaining sensing performance under humid conditions is a major challenge for semiconducting metal oxide‐based chemiresistors. To assess this, bare, Au‐, and Pd‐SnO_2_ HBNCs were exposed to 5 ppm CH_3_COCH_3_ at 300 °C under varying relative humidity (RH) levels from 0% to 80% (Figure S14, Supporting Information). Humidity degrades sensor performance by competing with gas molecules for adsorption sites, reducing sensitivity and slowing response time.^[^ ^46^ ^]^ As RH increases, water molecules accumulate on the surface, forming additional layers over the initial physisorbed layer—a phenomenon known as water poisoning.^[^ ^47^, ^48^ ^]^ Consequently, the base resistance and gas response of all sensors progressively declined with increasing RH. To evaluate robustness in high humidity environments, SnO_2_ HBNCs‐based sensors were tested for nine cyclic responses to seven gases at 5 ppm under 80% RH conditions. The results, summarized in Figure 3d–f and shown in Figure S15 (Supporting Information), demonstrate the impact of increased humidity compared to the dry condition (Figure 3a–c). Although responses under humid conditions were reduced, the trends in response magnitude and response time were largely maintained. Notably, the response to C_5_H_8_ remained relatively high, which is detailed in the section 2.5.

The standard deviation of measurement sets was calculated and is presented in the insets of Figure 3a–f. To provide an intuitive assessment of the deviation from the mean response for each gas, the CV was determined using the following equation

(1) $$\mathrm{CV}\left(\%\right)=\left(\mathrm{standard}\mathrm{deviation}/\mathrm{mean}\right)\times 100$$

Notably, none of the CV values exceeded 5% for any of the sensors, underscoring the exceptional stability of SnO_2_ HBNC‐based gas sensors under both dry and 80% RH conditions, as illustrated in Figure 3g. This consistency indicates highly reliable and repeatable sensor performance over time, even after multiple exposures to various gases. In comparison, commercial gas sensors often exhibit deviations ranging from 16.7% to 41%, while high‐accuracy sensors typically achieve deviations below 10%.^[^ ^49^ ^]^ The benchmark for ensuring minimal environmental or operational impact on sensor accuracy is a CV below 5%. The outstanding reliability of the fabricated sensors highlights their strong potential for practical applications beyond benchtop validation.^[^ ^50^ ^]^

### 2.4 Detection Limit of SnO_2_ HBNCs to Seven Gases

**Figure 4:**
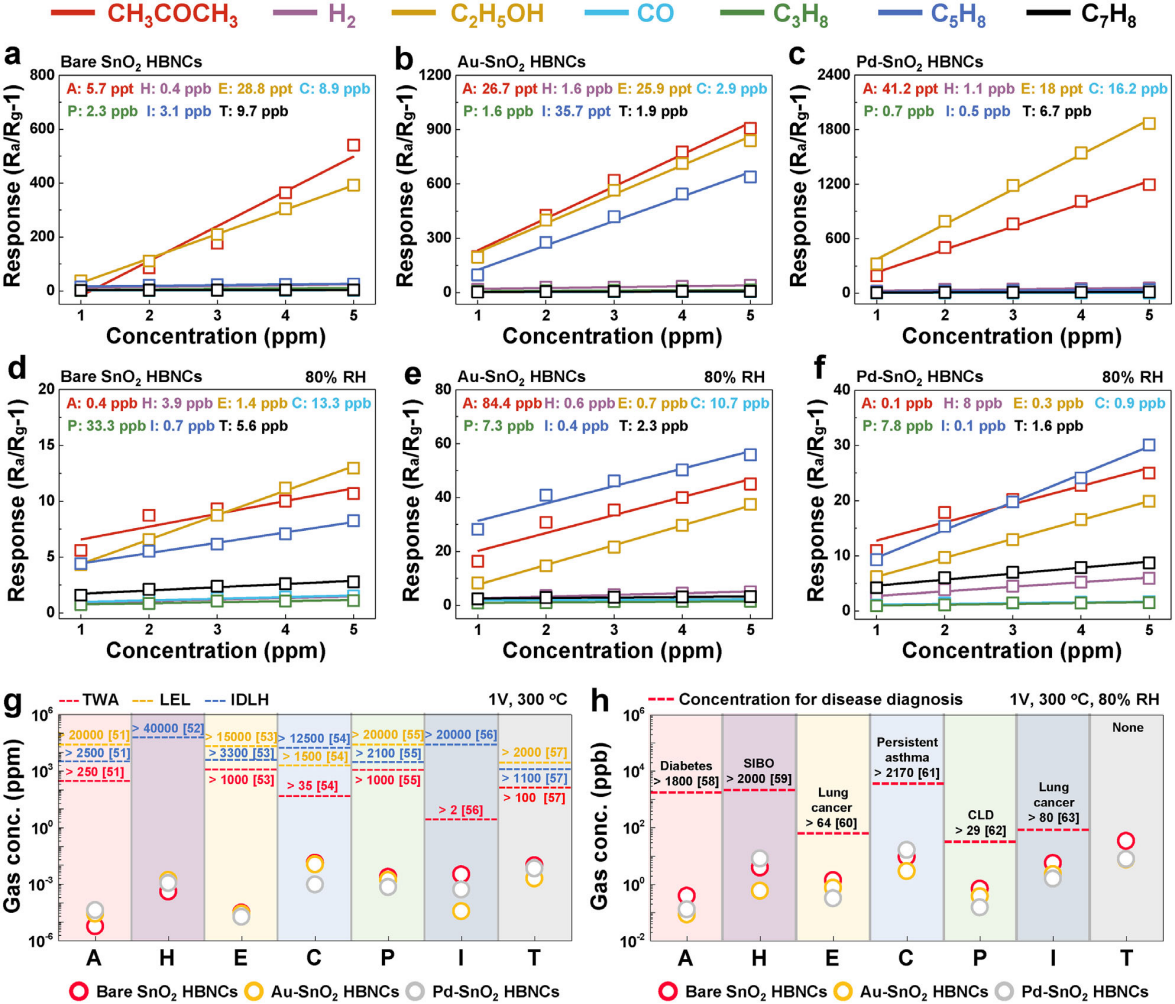
Reliability of the sensors with ultralow theoretical detection limits. Linear fitting graphs for 1–5 ppm of seven gases for a) bare, b) Au‐, and c) Pd‐SnO_2_ HBNCs under dry conditions, and d) bare, e) Au‐, and f) Pd‐SnO_2_ HBNCs under 80% RH conditions (A: acetone, H: hydrogen, E: ethanol, C: carbon monoxide, P: propane, I: isoprene, and T: toluene). g) Summary graph of concentrations relative to the detection limit (DL), time‐weighted average (TWA), lower explosive limit (LEL), and immediately dangerous to life or health (IDLH) levels, and h) concentrations relevant for disease diagnosis of seven gases for bare, Au‐, and Pd‐SnO_2_ HBNCs.

To further investigate the theoretical detection limits (DL) of each sensor, controlled concentrations of seven gases were tested under both dry (**Figure** **4**a–c) and 80% RH conditions (Figure 4d–f). All sensors exhibited a linear response to increasing concentrations, as detailed in Figures S16 and S17 (Supporting Information). The DL calculation methods are outlined in the Experimental Section. For example, under dry conditions, CH_3_COCH_3_ and C_2_H_5_OH were detected at the parts‐per‐trillion (ppt) level, while the other gases were detected at the parts‐per‐billion (ppb) level. In humid conditions, DL values decreased due to water vapor hindering effective gas–sensor surface interactions. Despite this, the sensors remained effective even at ultralow (sub‐ppb) concentrations.

DL is a critical parameter for assessing sensor applicability in daily use, with several key benchmarks highlighted in Figure 4g.^[^ ^51^, ^52^, ^53^, ^54^, ^55^, ^56^, ^57^ ^]^ The time‐weighted average (TWA) assesses long‐term exposure risks over an 8‐h workday or 40‐h workweek. The lower explosive limit (LEL) defines the lowest gas concentration forming a combustible mixture in air. The immediately dangerous to life or health (IDLH) threshold indicates exposure levels posing immediate threats to life or permanent health risks. The fabricated sensors achieved exceptionally low DL values, well below TWA, LEL, and IDLH thresholds, demonstrating their suitability for early detection of hazardous gases in occupational safety and environmental monitoring. Furthermore, sub‐ppb detection capability enables applications in diagnosing diseases such as diabetes, small intestinal bacterial overgrowth, lung cancer, persistent asthma, and chronic lung disease, where biomarkers in human exhaled gases can be reliably detected (Figure 4h).^[^ ^58^, ^59^, ^60^, ^61^, ^62^, ^63^ ^]^

The exceptional performance of the fabricated sensors, characterized by their stability and low DL, underscores their potential for diverse applications, including air quality monitoring, industrial safety (e.g., detecting hazardous gas leaks), environmental pollution control (e.g., monitoring volatile organic compounds), and medical diagnostics (e.g., analyzing biomarkers in exhaled breath for disease detection).

### 2.5 Identifying Sensing Patterns via Feature Visualization

**Figure 5:**
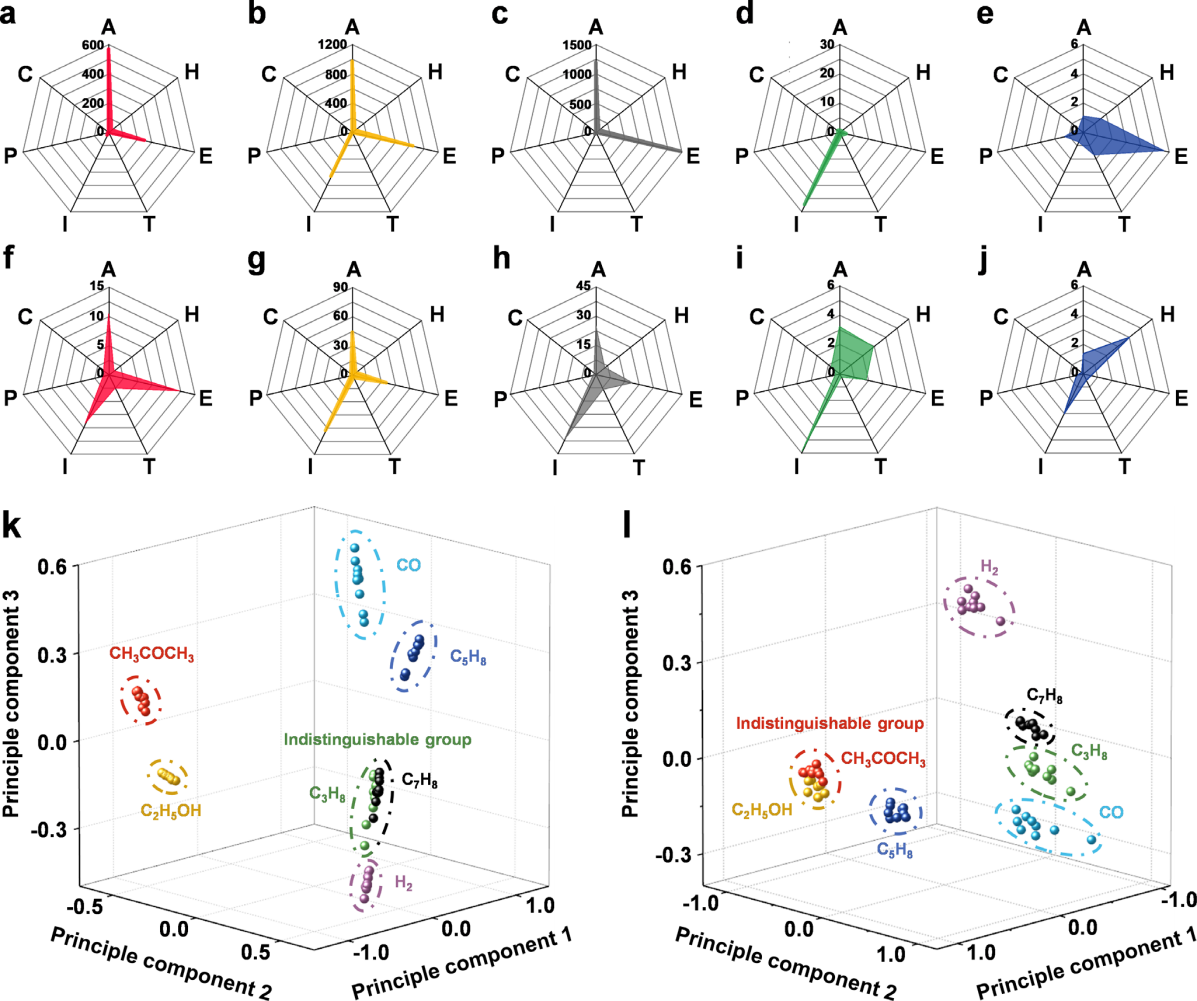
Radar plots and 3D PCA results for seven gases. Response radar plots for seven gases at 5 ppm and 300 °C under dry conditions: a) bare SnO_2_ HBNCs, b) Au‐SnO_2_ HBNCs, and c) Pd‐SnO_2_ HBNCs. Selectivity radar plots for seven gases at 5 ppm and 300 °C under dry conditions: d) Au‐SnO_2_ HBNCs/bare SnO_2_ HBNCs and e) Pd‐SnO_2_ HBNCs/bare SnO_2_ HBNCs. Response radar plots for seven gases at 5 ppm and 300 °C under 80% RH: f) bare SnO_2_ HBNCs, g) Au‐SnO_2_ HBNCs, and h) Pd‐SnO_2_ HBNCs under 80% RH. Selectivity radar plots for seven gases at 5 ppm and 300 °C under 80% RH: i) Au‐SnO_2_ HBNCs/bare SnO_2_ HBNCs and j) Pd‐SnO_2_ HBNCs/bare SnO_2_ HBNCs. 3D PCA results based on the response, response time, and recovery time from nine repeated measurements for seven gases at 5 ppm and 300 °C for three samples under k) dry and l) 80% RH conditions.

Achieving selective gas sensing performance remains a challenge. To address this, radar plots of bare, Au‐, and Pd‐SnO_2_ HBNCs were analyzed for responses to seven gases (**Figure** **5**a‐j). All sensors exhibited high sensitivity to CH_3_COCH_3_ and C_2_H_5_OH but low sensitivity to H_2_, CO, C_3_H_8_, and C_7_H_8_ (Figure 5a–c,f–h, respectively), reflecting differences in chemical interactions. The strong response to CH_3_COCH_3_ and C_2_H_5_OH stems from their high adsorption energy on SnO_2_, which facilitates oxidation with pre‐adsorbed oxygen.^[^ ^64^ ^]^ Their polar functional groups—carbonyl in CH_3_COCH_3_ and hydroxyl in C_2_H_5_OH—further enhance charge transfer. In contrast, H_2_, CO, C_3_H_8_, and C_7_H_8_ exhibit weak physisorption and lower oxidation efficiency, resulting in poor sensing responses.^[^ ^65^, ^66^, ^67^, ^68^ ^]^

Notably, Au‐SnO_2_ HBNCs showed enhanced selectivity for C_5_H_8_ due to Au's catalytic conversion of C_5_H_8_ into acetaldehyde (Figure 5b,d).^[^ ^44^ ^]^ Under 80% RH, all sensors maintained high C_5_H_8_ sensitivity, while trends for other gases remained consistent with dry conditions. This behavior is linked to polarity‐dependent interactions. Polar gases (CH_3_COCH_3_, C_2_H_5_OH, CO) interact strongly with hydroxyl groups, leading to competitive adsorption that hinders oxidation.^[^ ^69^ ^]^ In contrast, non‐polar gases (C_5_H_8_, C_3_H_8_, C_7_H_8_, H_2_) exhibit weaker hydroxyl interactions, making them less humidity‐dependent.^[^ ^70^ ^]^ Among them, C_5_H_8_ reacts efficiently due to its C═C bonds, which promote charge transfer.^[^ ^71^ ^]^ Meanwhile, C_3_H_8_ (C─C single bonds) and C_7_H_8_ (aromatic ring) are chemically stable, while a small size and high diffusivity of H_2_ limit its adsorption, resulting in low sensitivity.^[^ ^72^, ^73^ ^]^ These findings highlight the structural advantages that enhance C_5_H_8_ selectivity under humid conditions.

To improve gas differentiation, 3D principal component analysis (PCA) was conducted, incorporating response and response/recovery times. PCA enhanced selectivity for five of the seven gases under dry and 80 RH conditions (Figure 5k,l). However, certain gases (e.g., CH_3_COCH_3_ vs C_2_H_5_OH, C_3_H_8_ vs CO, C_3_H_8_ vs C_7_H_8_) remained difficult to distinguish due to similar chemical properties (Figure S18, Supporting Information).^[^ ^74^, ^75^ ^]^ Further analysis across 1–50 ppm via PCA (Figures S19 and S20, Supporting Information) confirmed persistent overlap, implying the need for advanced classification methods.

### 2.6 Preprocessing and Data Augmentation for Deep Learning

Distinguishing complex gas response patterns remains challenging for conventional machine learning methods, particularly when PCA fails to separate gas classes effectively. In such cases, deep learning offers a powerful alternative by learning hierarchical feature representations from large‐scale datasets. However, deep learning models typically require substantial training data to achieve high generalization performance. To address this, we applied extensive data augmentation techniques to enhance dataset diversity and improve model robustness.

Studies have shown that CNN‐based models trained with augmented datasets achieve superior classification accuracy and generalization. Aquino et al. (2017) demonstrated that data augmentation significantly improved CNN model performance, consistently outperforming models trained on raw data.^[^ ^76^ ^]^ Similarly, Poojary et al. (2021) found that fine‐tuning pre‐trained CNN models (e.g., VGG16 and ResNet50) with augmentation led to notable accuracy improvements, reinforcing its importance in deep learning applications.^[^ ^77^ ^]^ Consistent with these findings, our results show that CNN models trained with synthetic spectrograms exhibit greater robustness to environmental fluctuations compared to models trained solely on experimental data. To improve generalization, we employed a two‐step data augmentation approach: 1) DTW‐based upsampling that standardizes gas response curve lengths while preserving intrinsic structure and 2) SpecAugment that introduces controlled spectral variations, reducing overfitting and enhancing feature learning. These augmentation techniques were carefully selected to ensure the expanded dataset retains real‐world sensor characteristics while providing sufficient diversity for deep learning training.

**Figure 6:**
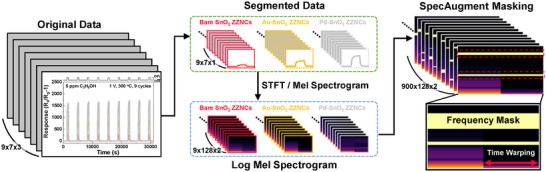
Data augmentation process using gas sensing properties data to seven gases for three samples.

A DTW‐based upsampling method was implemented to address the challenge of varying curve lengths across the seven gas types (**Figure** **6**). This technique dynamically aligns sequences of differing lengths by stretching or compressing the time axis to minimize alignment distances, thereby ensuring temporal consistency while preserving the intrinsic structure of the data.^[^ ^21^ ^]^

The individual curve length was set to 2048 points to meet the efficiency requirements of FFT algorithms, which perform optimally with data lengths that are powers of two. This choice ensured computational efficiency and preserved the integrity of the sensor data. To standardize total data length across all gas types, a fixed length of 18432 points was established, corresponding to nine response curves, each comprising 2048 points (2048 × 9 = 18432). This configuration supported uniform application of spectral transformation techniques, such as the short‐time Fourier transform (STFT) and mel‐spectrograms, which require consistent input lengths for optimal parameter tuning. Furthermore, the 18432‐point total accommodated C_2_H_5_OH, the gas with the longest recorded sensor data length of 17018 points, avoiding truncation or data loss.

The upsampled time‐series data were subsequently transformed into spectrograms for feature extraction. Spectral features were derived using STFT and mel‐spectrograms, with parameters optimized for resolution, including a 4096 Hz sampling rate, a 2048‐point FFT window size, and 128 mel filter banks. Log‐scale normalization was applied to highlight relative spectral differences and standardize data distribution, enhancing the compatibility of the data with deep learning models.

To address variability in real‐world sensor data caused by environmental fluctuations, additional data augmentation techniques were applied to improve model generalization. In addition to conventional methods such as noise addition, scaling, and shifting, SpecAugment—a technique originally developed for speech recognition—was utilized. This approach preserved the temporal characteristics of time‐series data while increasing dataset diversity, significantly enhancing generalization capabilities of the model.^[^ ^78^ ^]^ Frequency masking (maximum width of 20) randomly blocked specific frequency bands, reducing the risk of overfitting to particular patterns. Time warping (deformation parameter of ±2) simulated temporal variations, reflecting real‐world sensor dynamics. Time masking was excluded due to the limited resolution along the time axis, ensuring data integrity. The combination of frequency masking and time warping effectively expanded dataset diversity, ensuring that the dataset was sufficiently large for deep learning models while maintaining the original distribution of gas sensor responses.

As a result, the original dataset—comprising seven gases, three catalysts, and nine response curves—was expanded by generating 100 additional data points per curve. This resulted in a final dataset of 21 classes (seven gases × three sensors), each represented by 909 samples, providing adequate training data for robust model learning. The dataset was randomly divided into 72% for training, 8% for validation, and 20% for testing to ensure balanced evaluation.

### 2.7 Evaluating Model Performance for Artificial Olfactory System

**Figure 7:**
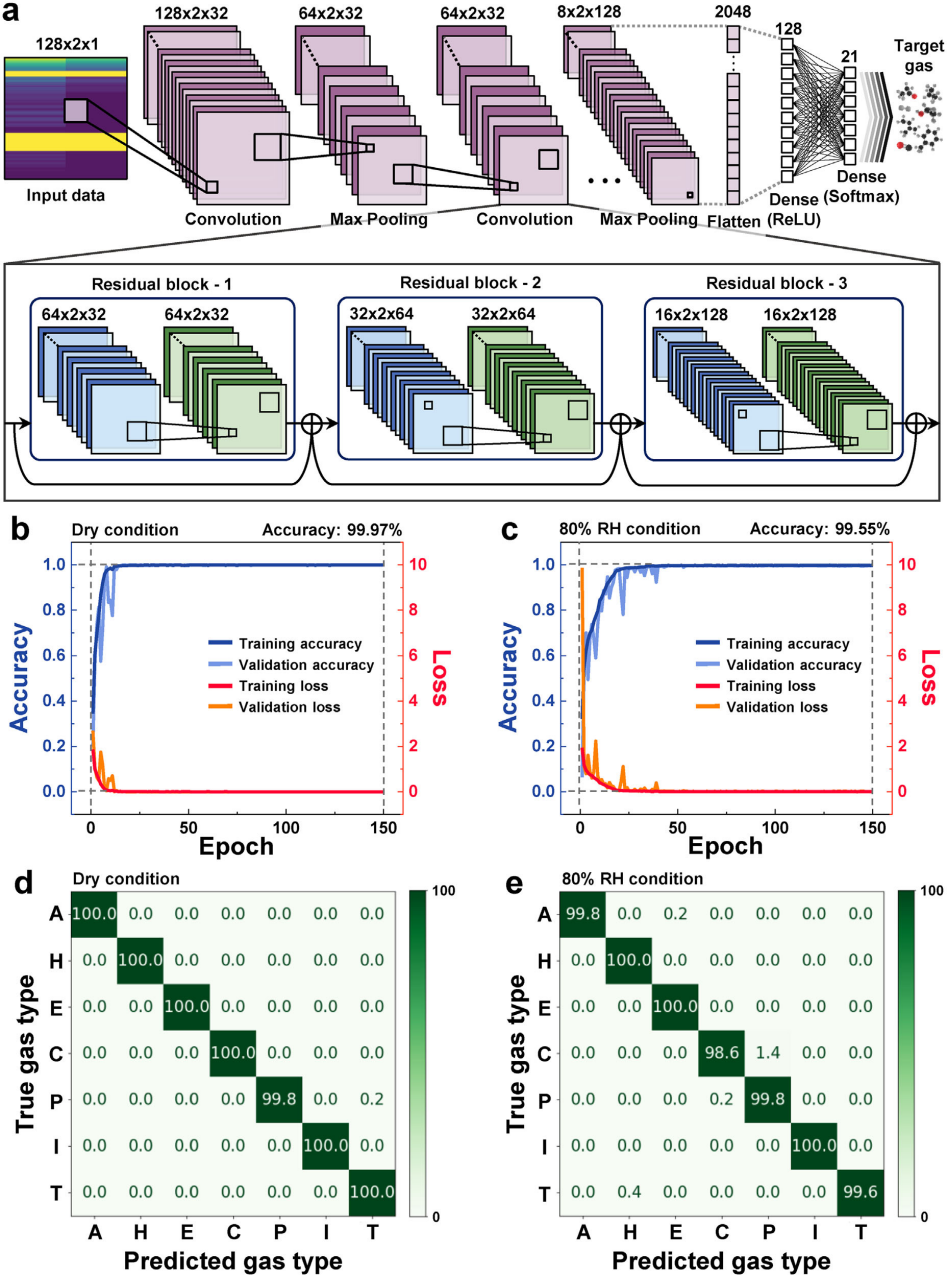
Classification accuracy using the ResNet model under dry and 80% RH conditions. a) Overview of the residual network (ResNet) model architecture. Accuracy and loss graphs for training and validation under b) dry and c) 80% RH conditions. Confusion matrices displaying prediction results for seven gases under d) dry and e) 80% RH conditions (A: acetone, H: hydrogen, E: ethanol, C: carbon monoxide, P: propane, I: isoprene, and T: toluene).

To classify the seven gases under varying conditions, we employed three deep learning architectures: CNN, CNN‐long short‐term memory (CNN‐LSTM) hybrid, and ResNet. CNN efficiently extracts spatial features from spectrogram representations of gas sensor signals. CNN‐LSTM integrates temporal dependencies, improving the modeling of dynamic time‐series data (Figure S21, Supporting Information).^[^ ^79^, ^80^, ^81^ ^]^ ResNet, utilizing residual connections, effectively addresses the vanishing gradient problem in deep networks, enabling the extraction of complex hierarchical features (**Figure** **7**a).^[^ ^82^ ^]^ The ResNet demonstrated superior robustness, particularly in handling sensor data affected by environmental variations such as high humidity (Figure 7b–e). A comparative analysis of these architectures was conducted to determine the most suitable model for accurate and reliable gas classification.

To ensure consistent evaluation of the three deep learning architectures, identical hyperparameters were used during training. A batch size of 64, an initial learning rate of 0.001, and 150 training epochs were selected based on a hyperparameter tuning process to optimize model accuracy. The ReduceLROnPlateau technique was employed to promote stable convergence, halving the learning rate if the validation loss did not improve over three consecutive epochs. This approach optimized model performance during the later stages of training.^[^ ^83^ ^]^

To prevent overfitting and enhance generalization to unseen data, multiple regularization techniques were implemented. Dropout layers were incorporated into all three architectures to reduce co‐adaptation among neurons, promoting the learning of independent and robust features. In the CNN model, dropout was applied after the fully connected layer at a rate of 0.3, balancing model capacity and generalization. The CNN‐LSTM model utilized dropout in both the LSTM units and dense layers, each with a rate of 0.3, ensuring stability in capturing spatial and temporal dependencies. The ResNet architecture incorporated dropout at a lower rate (0.2) in its dense layers, reflecting its built‐in regularization through residual connections. These residual connections mitigate the vanishing gradient problem, ensuring stable learning in deep networks while maintaining high generalization performance with a reduced dropout rate.

Batch normalization was extensively applied across the convolutional layers of all models to stabilize training dynamics and improve convergence. Data augmentation techniques, including SpecAugment, were employed to introduce controlled variations in the training data, further enhancing model generalization. To validate model robustness, five‐fold cross‐validation was conducted to ensure that the models were not overly dependent on specific subsets of the training data.

The classification performance of ResNet, CNN, and CNN‐LSTM was evaluated under both dry and 80% RH conditions (**Table** **1**). ResNet consistently outperformed the other architectures, achieving near‐perfect accuracy (99.97%) under dry conditions with a loss of only 0.0005. Even under 80% RH, ResNet demonstrated robust performance, attaining an accuracy of 99.55% and a loss of 0.0181. These results emphasize the importance of effective data preprocessing, augmentation, and robust model selection in maintaining classification performance despite temporal and environmental variations. The residual connections in ResNet played a critical role in extracting complex features from the spectrograms, thereby enhancing both reliability and interpretability. These findings are consistent with previous studies highlighting the effectiveness of mel spectrogram representations combined with deep learning for gas classification tasks.^[^ ^13^ ^]^ Although CNN and CNN‐LSTM performed well under dry conditions, their accuracy and robustness slightly declined at higher humidity levels. The CNN architecture efficiently extracted spatial features, while the CNN‐LSTM model adapted to the temporal variations induced by high humidity (Figure S22, Supporting Information). However, both architectures lagged behind ResNet in terms of overall accuracy and robustness (**Table** **2**). To further assess generalization capabilities, five‐fold cross‐validation was conducted. ResNet achieved the highest average accuracy under dry conditions (99.95%), outperforming CNN (98.37%) and CNN‐LSTM (96.80%). At 80% RH, ResNet maintained superior performance, with an average accuracy of 99.07%, while CNN and CNN‐LSTM achieved 97.83% and 96.56%, respectively. These results highlight the ability of ResNet to consistently adapt to diverse environmental conditions and its superior feature extraction capabilities.

**Table 1:** Comparison with three learning models. Summary of the test results for the CNN, CNN‐LSTM, and ResNet models for seven gases under dry and 80% RH conditions.

Conditions	Model	Precision	Recall	F1‐Score	Accuracy
Dry	CNN	1.0000	1.0000	1.0000	99.81%
	CNN‐LSTM	0.9872	0.9872	0.9872	97.82%
	ResNet	1.0000	1.0000	1.0000	99.97%
80% RH	CNN	0.9961	0.9961	0.9961	99.60%
	CNN‐LSTM	0.9801	0.9801	0.9801	96.38%
	ResNet	0.9974	0.9974	0.9974	99.55%

**Table 2:** Five‐fold cross‐validation of three models. Summary of five‐fold cross‐validation results of CNN, CNN‐LSTM, and ResNet models for seven gases under dry and 80% RH conditions.

Fivefold cross‐validation			Fold 1	Fold 2	Fold 3	Fold 4	Fold 5	Average
Dry condition	CNN	Accuracy	99.76%	99.73%	99.68%	95.54%	97.11%	98.37%
		Loss	0.0071	0.0081	0.0120	0.1121	0.0749	0.0429
	CNN‐LSTM	Accuracy	98.40%	97.59%	94.31%	98.48%	95.20%	96.80%
		Loss	0.0557	0.0739	0.1734	0.0577	0.1460	0.1014
	ResNet	Accuracy	99.97%	99.97%	99.94%	99.92%	99.94%	99.95%
		Loss	0.0009	0.0006	0.0012	0.0018	0.0008	0.0011
80% RH condition	CNN	Accuracy	95.67%	98.11%	97.14%	98.48%	99.73%	97.83%
		Loss	0.1408	0.0803	0.1025	0.0615	0.0152	0.0801
	CNN‐LSTM	Accuracy	97.17%	96.20%	97.17%	97.77%	94.49%	96.56%
		Loss	0.0973	0.1244	0.1077	0.0791	0.1586	0.1134
	ResNet	Accuracy	99.44%	99.76%	96.59%	99.63%	99.89%	99.07%
		Loss	0.0258	0.0068	0.1278	0.0127	0.0063	0.0359

Model storage efficiency and computational cost are critical factors for practical deployment alongside classification performance. Table S1 (Supporting Information) summarizes the model size, inference time, and FLOPs for each architecture, highlighting their trade‐offs. For example, CNN is the most lightweight, with a small model size (4.16 MB) and lowest computational cost (7.83 MFLOPS), making it ideal for resource‐constrained applications. CNN‐LSTM, incorporating temporal dependencies, has the highest computational cost (28.83 MFLOPS) and longest inference time (84.51 ms). ResNet, while slightly larger than CNN, achieves a balance between efficiency and performance, with 27.57 MFLOPS and a 73.70 ms inference time, offering strong feature extraction capabilities while remaining computationally manageable. These results indicate that ResNet is the well‐rounded choice, providing high classification accuracy with reasonable storage and computational demands, making it suitable for real‐world deployment.

Additionally, the low CV values (<5%) in sensor data significantly contributed to model stability and accuracy. Gracheva et al. (2021) demonstrated that reducing CV in neural architecture search improves model reliability by minimizing variability in initialization and feature extraction.^[^ ^84^ ^]^ Similarly, in this study, the low CV of sensor data ensured a consistent input distribution, enhancing the model's ability to generalize effectively to unseen conditions. This consistency reinforced ResNet's sustained high classification performance, even in challenging environments such as high humidity.

Overall, these findings demonstrate the successful application of deep learning techniques to address the challenges of gas classification in complex and variable environments. The integration of reliable SnO_2_ HBNCs‐based gas sensors with advanced deep learning architectures provides a robust framework for developing artificial olfactory system capable of precise gas detection across a wide range of real‐world conditions. The high uniformity and low variability of the sensor data played a crucial role in achieving these strong performances, underscoring the importance of high‐quality input data in developing data‐driven sensor systems.

## 3 Conclusion

This study presents a data‐centric approach to advancing AI‐assisted electronic nose systems by integrating nanostructure design with deep learning. By fabricating highly reliable SnO_2_ HBNCs functionalized with Au and Pd nanocatalysts, we developed a robust sensing platform optimized for gas diffusion, reaction kinetics, and long‐term stability. Systematic aging procedures enhanced sensor reliability, ensuring reproducible datasets with a CV below 5%. The integration of these high‐performance sensors with deep learning, particularly ResNet model, achieved over 99.5% classification accuracy even under 80% RH. Additionally, the sub‐ppb detection limits of the sensor platform highlight its potential for real‐world applications in environmental monitoring, medical diagnostics, and industrial safety. This study features the importance of a holistic approach that combines material design with AI‐driven data analysis, establishing a foundation for future advancements in electronic nose technology and artificial olfaction that expand more reliable and adaptable solutions across diverse applications.

## 4 Experimental Section

### Preparation of SnO_2_ HBNCs‐Gas Sensors

Gold IDEs were fabricated using photolithography and e‐beam deposition techniques. The SiO_2_/Si substrate was sequentially cleaned in a sonicator with acetone, isopropanol, and deionized water for 5 min per step. A uniform layer of photoresist (LOR 5A, MicroChem Corporation) was spin‐coated onto the SiO_2_/Si substrate at 3000 rpm for 30 s and baked at 190 °C for 5 min. A second layer of photoresist (AZ GXR 601, AZ Electronic Materials) was then applied using the same spin‐coating conditions and baked at 150 °C for 1 min. IDE patterns were formed using a mask aligner system (MDA‐400S, MIDAS System). The patterned photoresist was developed using AZ 300MIF (MicroChemicals) and rinsed with deionized water. After photolithography, Au/Pt/Cr (100 nm/70 nm/30 nm) layers were deposited onto the patterned SiO_2_/Si substrate using an electron beam evaporator (EBX‐1000, ULVAC). To pattern the Au/Pt/Cr layers into IDEs, the remaining photoresist was removed with mr‐Rem 700 (micro resist technology), and the substrate was cleaned with ethanol and deionized water. The resulting IDEs consisted of 16 fingers, with a spacing of 20 µm between them.

Subsequently, the Au‐IDE‐patterned SiO_2_/Si substrate was loaded into an e‐beam evaporator (Korea Vacuum), and a sequential deposition process was performed to control the number of SnO₂ (99.99%, Taewon Scientific Co.) HBNC layers. First, SnO_2_ inclined nanocolumns were deposited onto the substrate at a tilt angle of 80°. The substrate was then rotated 180° for an additional deposition in the opposite direction. Then, the repeated deposition was conducted sequentially to fabricate 3‐, 5‐, and 7‐layered SnO_2_ HBNCs. For Au (99.99%, Taewon Scientific Co.) and Pd (99.98%, Taewon Scientific Co.) NP decoration, Au and Pd films with varying thicknesses (0.5, 1, 3, 5, and 10 nm) were deposited onto the surface in its original position (0°), as shown in Figure 1b. Finally, all samples were annealed at 500 °C for 1 h in an ambient air, transforming the metal films into nanoparticles and resulting in Au‐ and Pd‐decorated SnO_2_ HBNCs. Additionally, SnO_2_ thin films were fabricated on the substrate at the original angle (0°).

### Characterization

The cross‐sectional and top‐view morphologies of the SnO_2_‐based gas sensors were examined using field‐emission scanning electron microscopy (FE‐SEM, JSM‐7610F‐Plus, JEOL) at an acceleration voltage of 15 kV and a working distance of 8 mm. Crystallographic properties were evaluated through XRD (Empyrean, PANalytical) using Cu‐Kα radiation (*λ* = 1.5418 Å) under a tube voltage of 40 kV and a current of 30 mA. Chemical interactions between the materials were investigated using XPS (NEXSA G2, Thermo Fisher Scientific) with monochromatic Al Kα radiation. Additional crystallographic analysis and characterization of the Au and Pd nanoparticles were performed using transmission electron microscopy (TEM, Technai G2 F20, FEI) at an acceleration voltage of 200 kV.

### Gas Sensing Measurement for Seven Gases

The gas‐sensing system comprised a compact probe chamber with a 12‐channel probe system (MPS6000, PHOCOS), a mass flow controller (PHOCOS) for precise gas concentration regulation, and a temperature controller (PHOCOS). The chamber had dimensions of 140 mm in width, 80 mm in length, and 40 mm in height. After the sensor resistance stabilized to its initial baseline value in air, target gases (CH₃COCH₃, C₂H₅OH, H₂, CO, C₃H₈, C₅H₈, and C₇H₈, all balanced with air, Gaschem Technology Co.) were alternately injected at a combined flow rate of 1000 sccm using an automated gas control system. The operating temperature was regulated with a silicon nitride heater and alumina pad, which allowed adjustments from 25 to 400 °C. Gas response measurements for the seven gases were conducted at a constant DC bias voltage of 1 V using a source meter (Keithley 238, KEITHLEY), with sensor resistance precisely recorded using a high‐density switch system (7001 Switch System, KEITHLEY) to enable simultaneous measurement of multiple sensors. Resistance changes were recorded at intervals of ≈0.6 s using I.V. Solution software to analyze the dynamic gas response.

### The Theoretical Detection Limit Calculations

The DL was determined using a signal‐to‐noise ratio of 3, as described by the following equations

(2) $$Vx^{2}=\sum {\left(y_{i}-y\right)}^{2}$$

(3) $$\mathrm{rms}\;=\sqrt{\frac{Vx^{2}}{N}}$$

(4) $$\mathrm{DL}=3\left(\frac{\mathrm{rms}}{\mathrm{slope}}\right)$$

Before exposure to each gas, ten consecutive resistance values were recorded to establish the baseline response of the sensor. These baseline values, where 𝑦_𝑖_ represents the measured response values prior to exposure to the target gas, were used to calculate the variation from the average response (𝑦), determined using a fifth‐order polynomial fitting curve. The root‐mean–square (rms) noise was calculated using Equation (3), where 𝑁 represents the number of data points used in the curve‐fitting procedure. Subsequently, the DL for the three samples exposed to the seven gases under both dry and 80% relative humidity (RH) conditions was calculated using Equation (4). The RMS noise and slope values employed in these calculations are summarized in Tables S2 and S3 (Supporting Information).

### Experimental Setup

All experiments were conducted using Google Colab, a cloud‐based platform offering access to GPU resources. The hardware environment included an NVIDIA Tesla T4 GPU with 16 GB of VRAM and Compute Capability 7.5, which facilitated the efficient training and inference of deep learning models. The software environment consisted of Python 3.10.12, TensorFlow 2.17.1, and key libraries such as NumPy 1.26.4, Pandas 2.2.2, Matplotlib 3.8.0, Librosa 0.10.2.post1, and Scikit‐learn 1.5.2. The dataset was stored in Google Drive and accessed directly within the Colab environment, ensuring seamless data loading and storage. Model checkpoints and logs were saved to the same directory to guarantee reproducibility. GPU acceleration was employed throughout the experiments, significantly reducing computation time for both training and evaluation.

### Data Preparation, Feature Extraction

The dataset consisted of sensor response curves measured for seven gases (CH_3_COCH_3_, H_2_, C_2_H_5_OH, CO, C_3_H_8_, C_5_H_8_, and C_7_H_8_) across three catalyst types (bare, Au, Pd), resulting in 21 unique gas‐catalyst combinations. For each combination, nine response curves were measured under controlled conditions, yielding a total of 189 curves. To ensure temporal consistency, the response curves were standardized to 2048 points using a DTW‐based upsampling technique. The standardized time‐series data were then transformed into spectrogram representations for feature extraction. STFT was applied with a 2048‐point FFT window size and a 4096 Hz sampling rate to capture the frequency‐domain features of the sensor signals. Furthermore, mel‐spectrograms were generated using 128 mel filter banks to emphasize perceptually significant frequency components. Both STFT and mel‐spectrograms were log‐scale normalized to standardize the amplitude distribution and highlight relative differences between features, thus improving the quality of the feature representation. These preprocessed spectrograms served as input for the deep learning models.

### Data Augmentation

To enhance the generalization capabilities of the models and simulate real‐world variability, spectrogram‐based data augmentation techniques were employed. These techniques effectively increased the diversity of the dataset while preserving the underlying structure of the original data. The augmentation process included frequency masking and time warping, which significantly expanded the dataset. For each of the nine response curves measured per gas‐catalyst combination, 100 additional samples were generated using these augmentation methods. Consequently, the final dataset comprised 909 samples per class, distributed across 21 gas‐catalyst classes.

### Deep Learning Model

Three deep learning architectures–CNN, CNN‐LSTM, and ResNet–were implemented to classify gas sensor data, with each model designed to address the unique challenges posed by spectrogram‐like input data and the temporal and spatial characteristics of sensor signals.

The ResNet architecture incorporated residual connections to mitigate the vanishing gradient problem and facilitate the learning of hierarchical features. As detailed in Table S4 (Supporting Information), the model consisted of three residual blocks with increasing filter sizes (32, 64, and 128). These residual blocks enabled the model to capture complex feature relationships while maintaining stable gradients during training. The network concluded with fully connected layers, including a dropout layer to prevent overfitting, and a softmax layer for classification.

The CNN architecture, described in Table S5 (Supporting Information), comprised three convolutional layers followed by fully connected layers. The convolutional layers employed ReLU activation and max pooling to extract spatial features, while batch normalization stabilized training by standardizing intermediate feature distributions. Although this baseline model efficiently processed spectrogram inputs, it exhibited limitations in capturing temporal dependencies.

The CNN‐LSTM model, as outlined in Table S6 (Supporting Information), extended the CNN architecture by integrating two LSTM layers to capture sequential patterns in the data. The model featured three convolutional layers, each followed by batch normalization and max pooling. The extracted feature maps were reshaped and passed through the LSTM layers, which effectively modeled temporal dependencies. Fully connected layers, including a dropout layer to mitigate overfitting, were used to generate the final classification output.

All models were trained using the Adam optimizer with a learning rate of 0.001 and categorical cross‐entropy loss. ReLU activation was applied to all hidden layers, while the softmax function was employed in the output layer to compute probability distributions across the 21 classes. Training was conducted over 150 epochs with a batch size of 64. The ReduceLROnPlateau callback dynamically adjusted the learning rate when the validation loss plateaued for three consecutive epochs, ensuring stable convergence. The models were evaluated on the test set, which comprised 20% of the total dataset. ResNet achieved the best performance, with an accuracy of 99.97% under dry conditions and 99.55% under 80% RH conditions. While CNN and CNN‐LSTM also delivered strong results, they exhibited slightly reduced robustness in high‐humidity environments compared to ResNet. These findings highlight the effectiveness of the preprocessing techniques, augmentation methods, and the proposed deep learning architectures in achieving reliable gas classification under varying conditions.

## Conflict of Interest

The authors declare no conflict of interest.

## Supporting information



Supporting Information

## Data Availability

The data supporting the study findings are available from the corresponding author upon reasonable request.
