# Supplementary material for: Artificial Olfactory System Enabled by Ultralow Chemical Sensing Variations of 1D SnO2 Nanoarchitectures
Source: Adv Sci (Weinh). 2025 May 3;12(25):2501293. doi: 10.1002/advs.202501293 (PMC12224986; doi:10.1002/advs.202501293)
Supplement: Supplementary file 1 — Supporting Information [file ADVS-12-2501293-s001.docx]

**Supporting Information**

**Artificial Olfactory System Enabled by Ultralow Chemical Sensing Variations of 1D SnO_2_ Nanoarchitectures**

*Yun-Haeng Cho, Dong-Su Kim, Jung Hwan Seo, Jae Han Chung, Zion Park, Ki Chang Kwon, Jae-Kwon Ko, Tae Won Ha, Jeong-O Lee, Gyu-Li Kim, Seong-Jun Ro, Hyojung Kim, Chil-Hyoung Lee*, Kwangjae Lee*, Young-Seok Shim*, and Donghwi Cho**

**Supporting Figures**


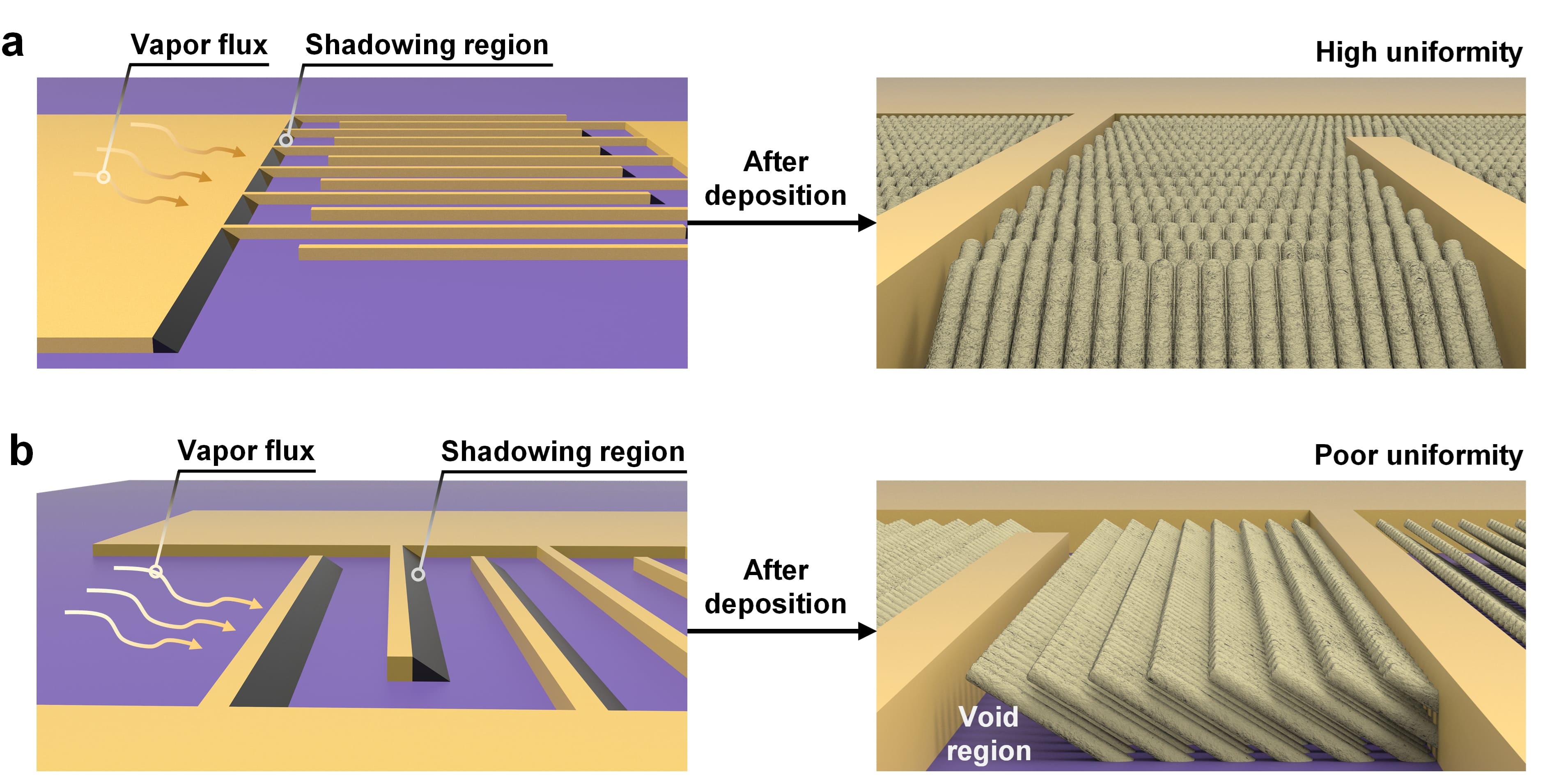


**Fig. S1.** Schematic illustrations of the deposited structure depending on the deposition direction. **a)** deposition parallel to the electrode fingers and **(b)** deposition perpendicular to the electrode fingers.


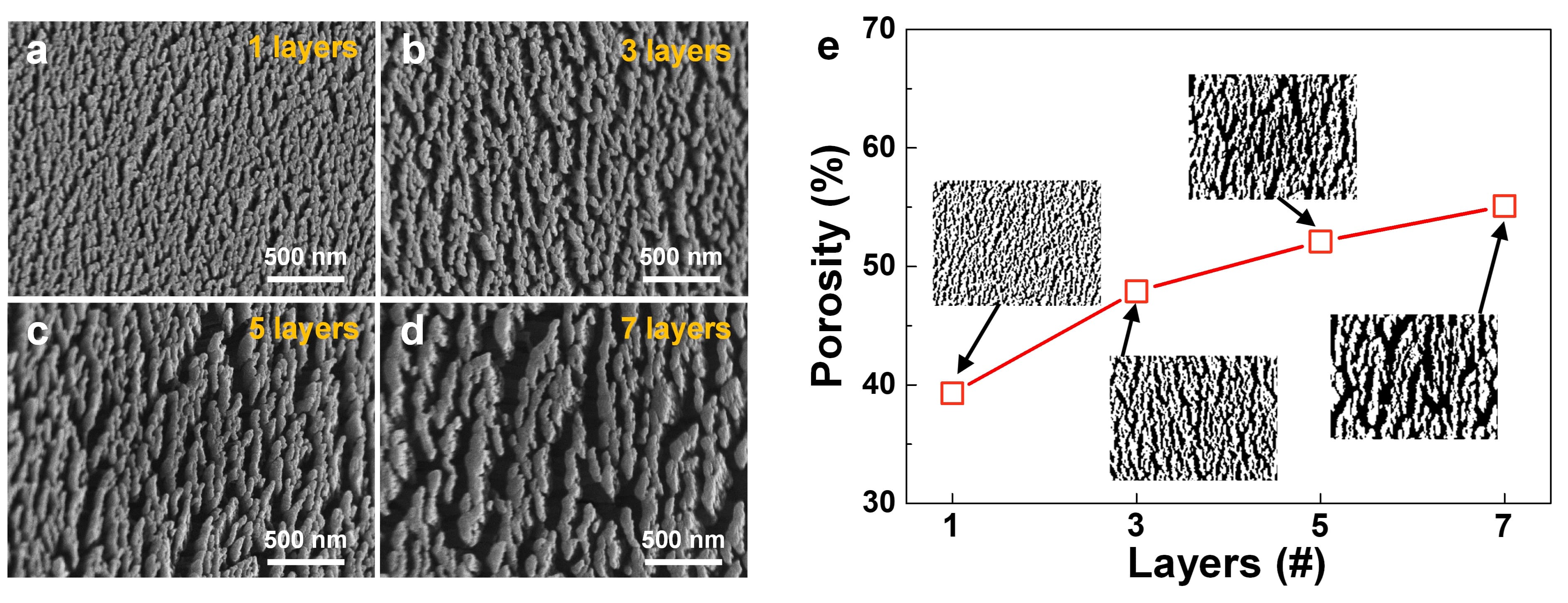
**Fig. S2.** Tilted-view SEM images and calculated porosity of SnO_2_ HBNCs. **a-d)** Tilted-view SEM images of 1-, 3-, 5-, and 7-layered SnO_2_ HBNCs. **e)** Calculated porosity of 1-, 3-, 5-, and 7-layered SnO_2_ HBNCs obtained using MATLAB. The inset displays the converted tilted-view SEM images processed in MATLAB.


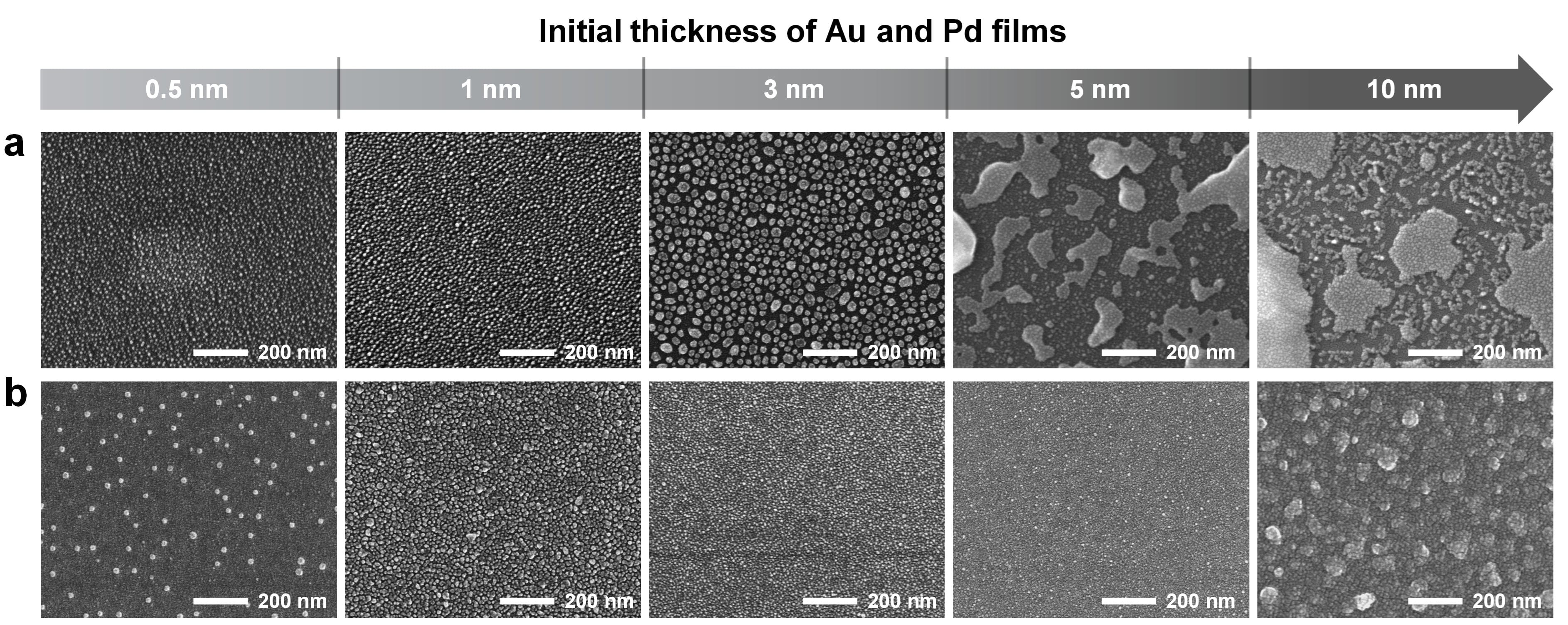
**Fig. S3.** Top-view SEM images of **(a)** Au and **(b)** Pd films with different initial thicknesses (0.5, 1, 3, 5, and 10 nm) on SiO_2_/Si substrate.


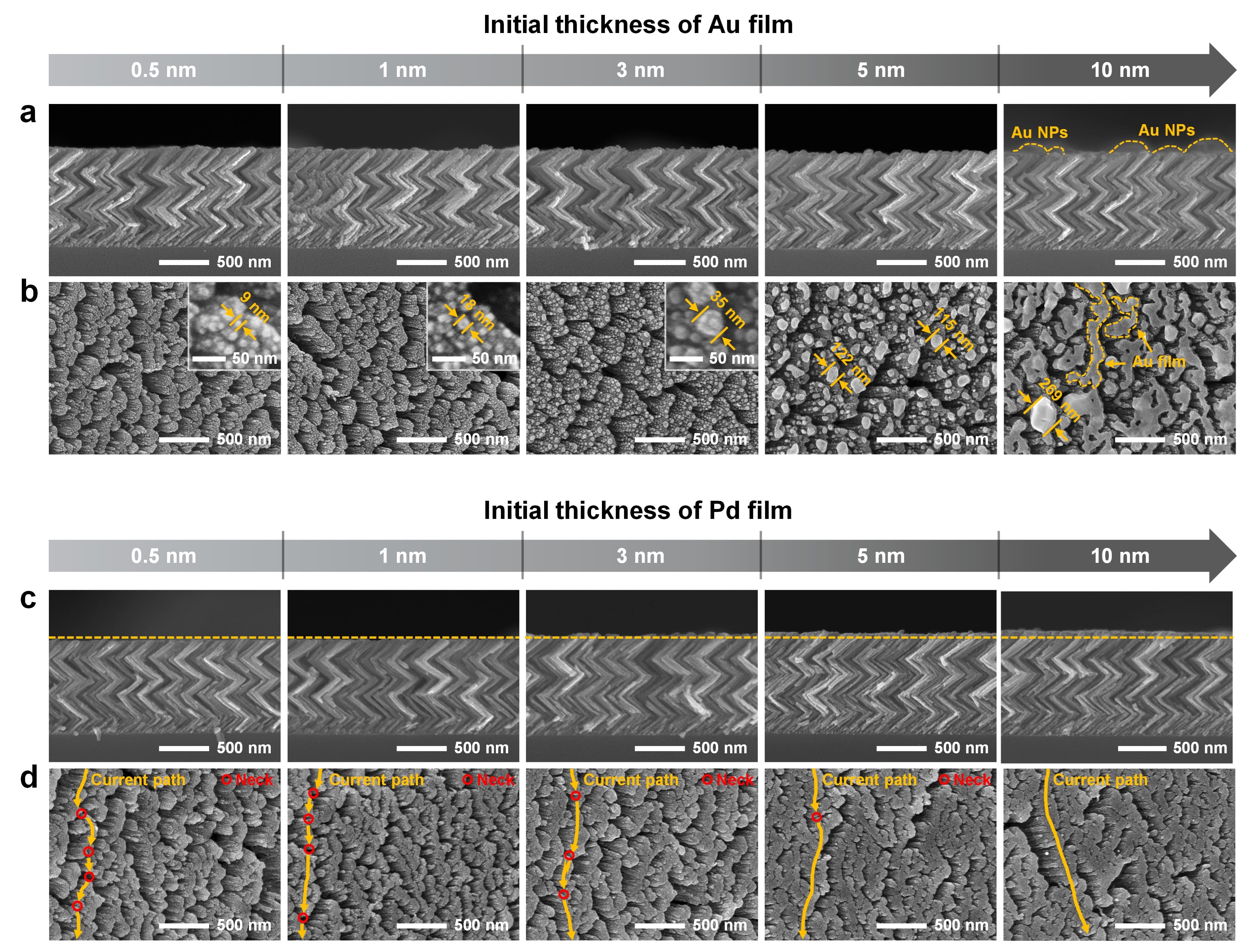


**Fig. S4.** Cross-sectional and top-view SEM images of **(a, b)** Au- and **(c, d)** Pd-decorated SnO_2_ HBNCs with different initial thickness of Au and Pd films: 0.5, 1, 3, 5, and 10 nm. The insets in **(c)** show the size of Au NPs in high magnification SEM image.


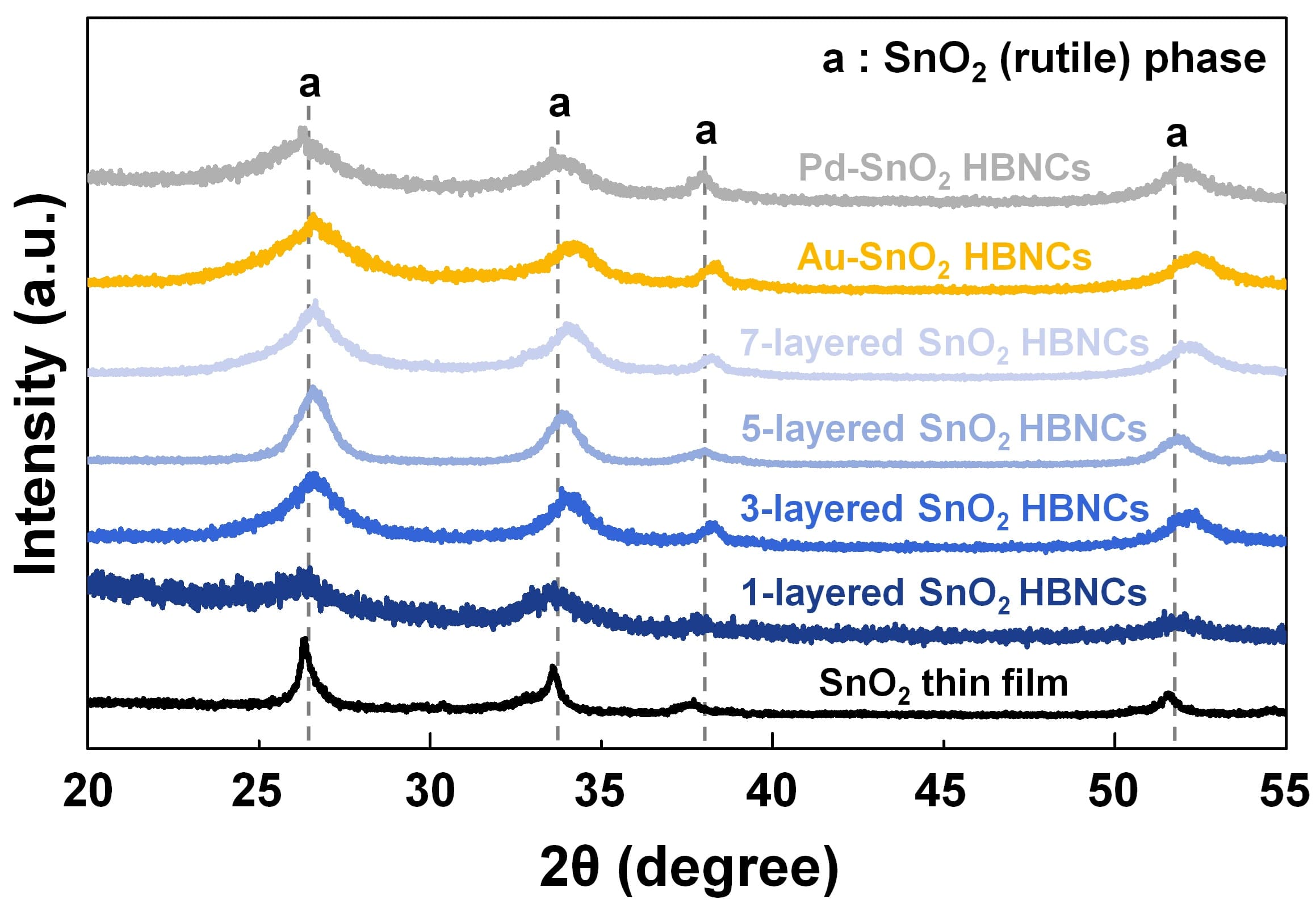


**Fig. S5.** XRD analysis results of the fabricated SnO_2_ HBNCs. XRD analysis data for SnO_2_ thin film, as well as 1-, 3-, 5-, and 7-layered SnO_2_ HBNCs, Au-SnO_2_ HBNCs, and Pd-SnO_2_ HBNCs.


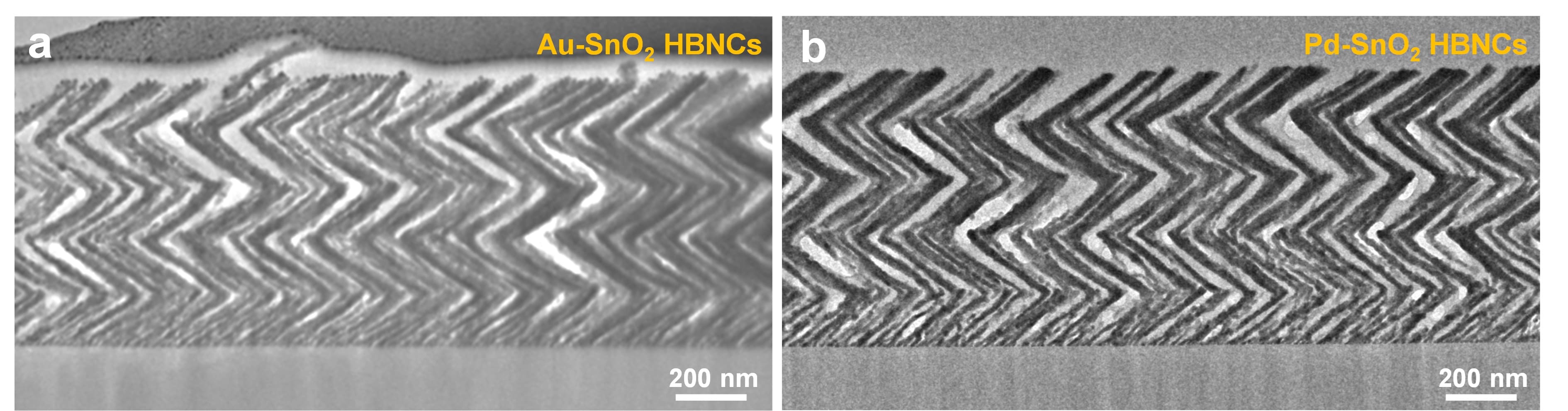


**Fig. S6.** HR-TEM images of **(a)** Au- and **(b)** Pd-SnO_2_ HBNCs, respectively.


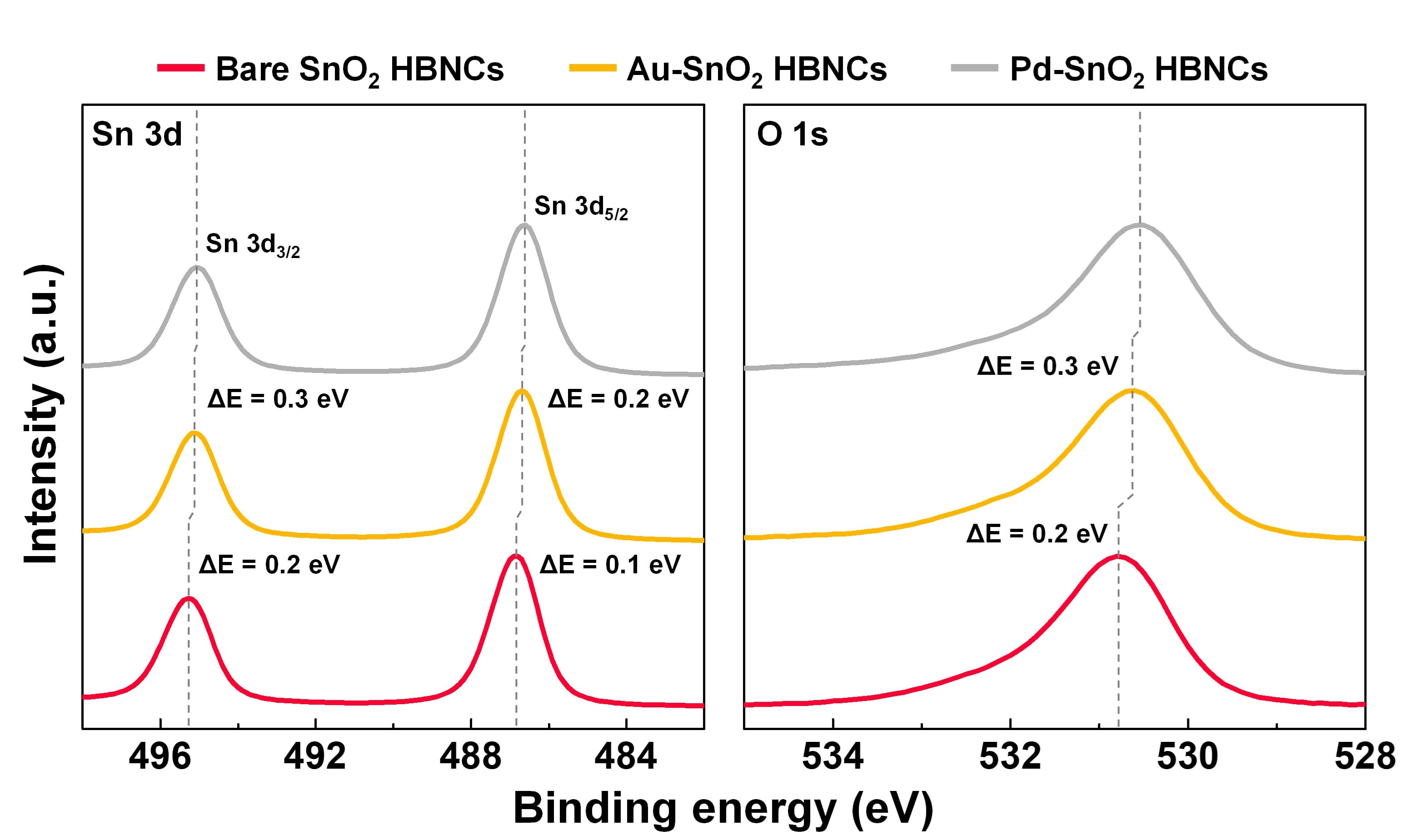


**Fig. S7.** XPS data for bare, Au-, and Pd-SnO_2_ HBNC.
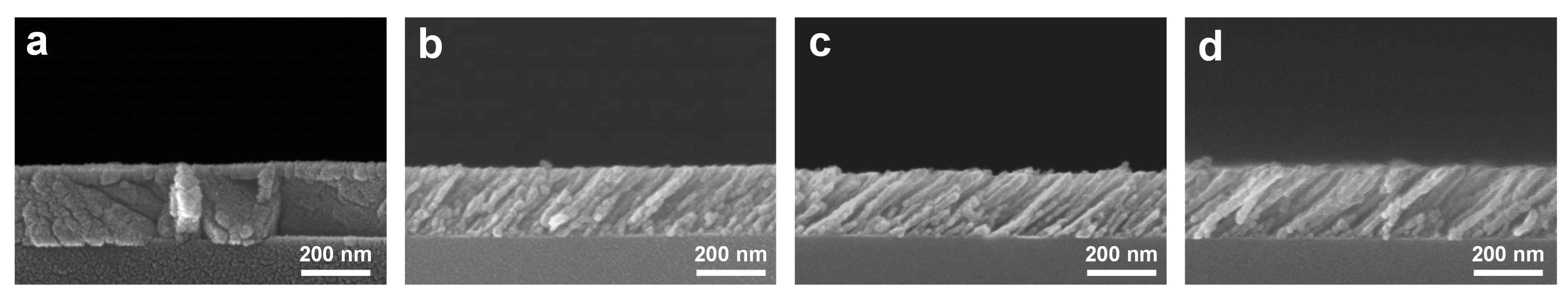


**Fig. S****8.** Cross-sectional SEM images of **(a)** SnO_2_ thin film and 1-layered SnO_2_ HBNCs deposited at **(b)** 75^o^, **(c)** 80^o^, and **(d)** 85^o^.


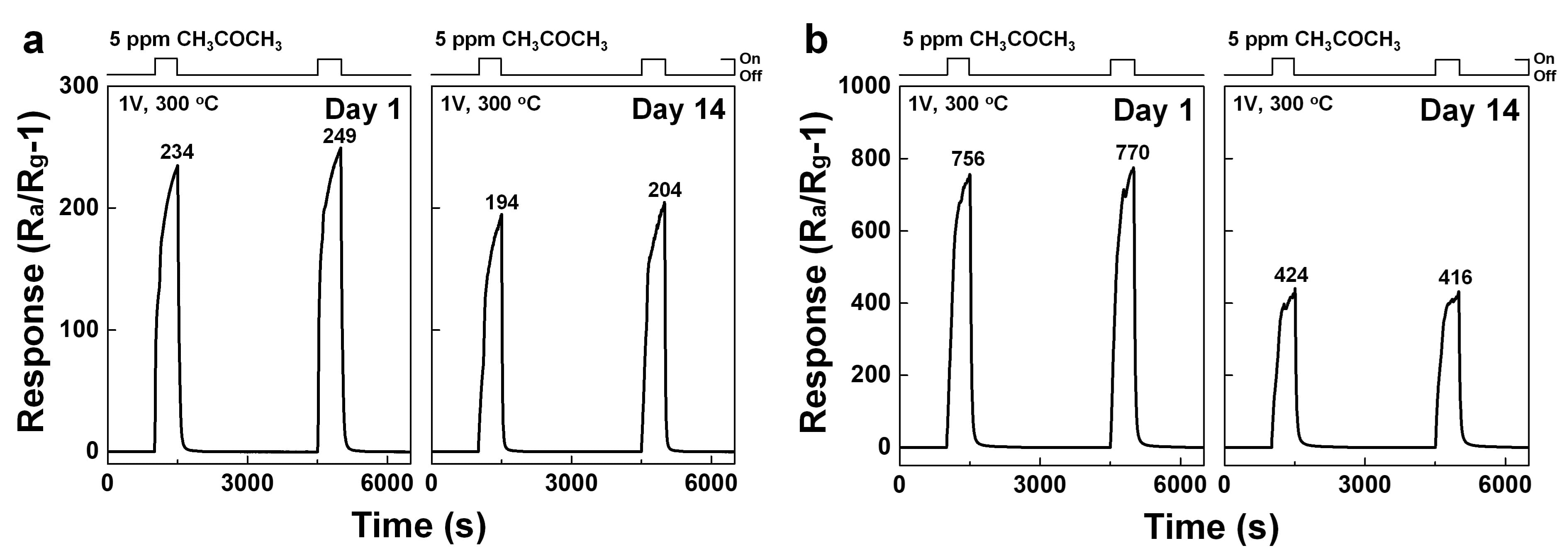


**Fig. S9.** Aging process of **(a)** 1- and **(b)** 5-layered SnO_2_ HBNCs deposited at 80^o^, monitored through measurements at 5 ppm CH_3_COCH_3_ and 300 ^o^C for 2 weeks.


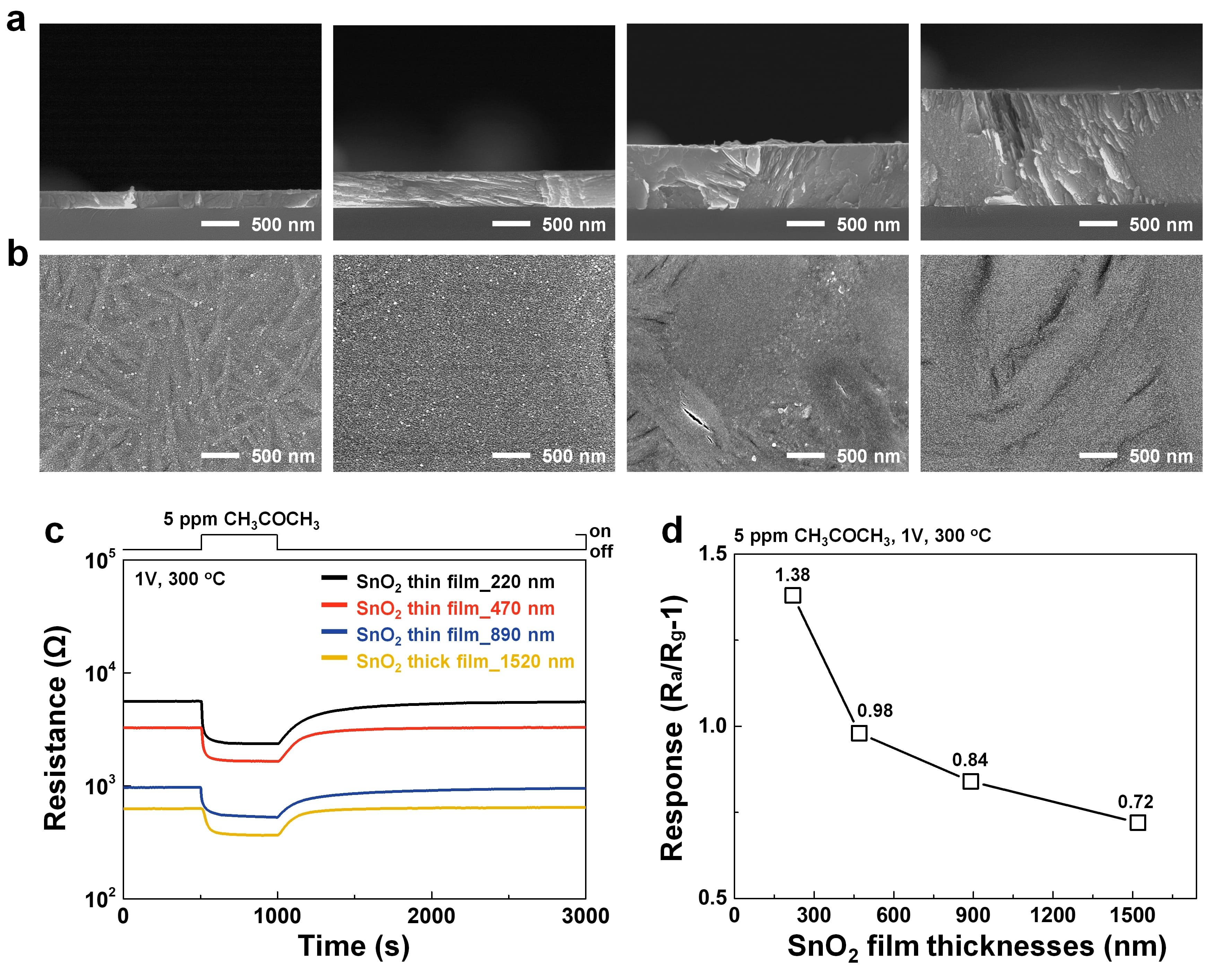


**Fig. S10**. **a)** Cross-sectional and **(b)** top-view SEM images of SnO_2_ film with varying thicknesses: 220, 470, 890, and 1520 nm. **c)** Response curves and **(d)** responses to 5 ppm CH_3_COCH_3_ at 300 ^o^C of SnO_2_ film as a function of thickness.


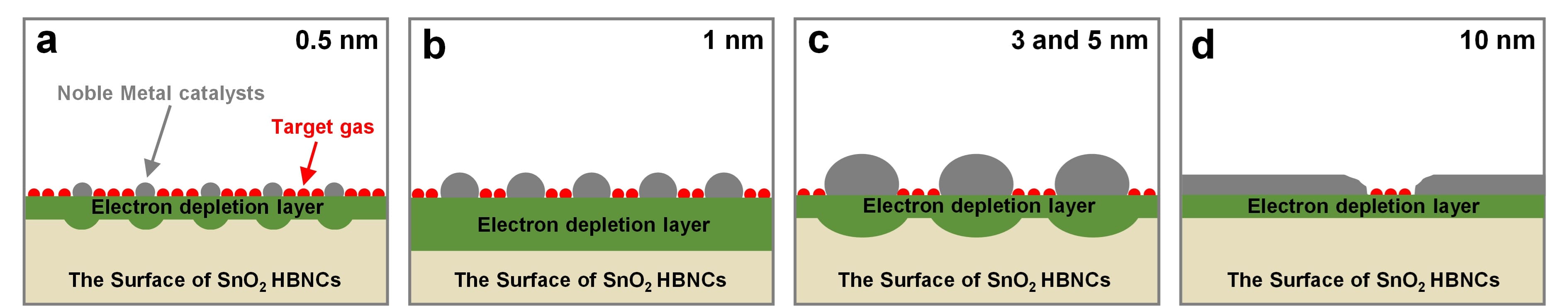


**Fig. S11.** Effect of the distribution of noble metal catalysts with varying initial thicknesses on the electron depletion layer: **(a)** 0.5 nm, **(b)** 1 nm, **(c)** 3 and 5 nm, and **(d)** 10 nm.


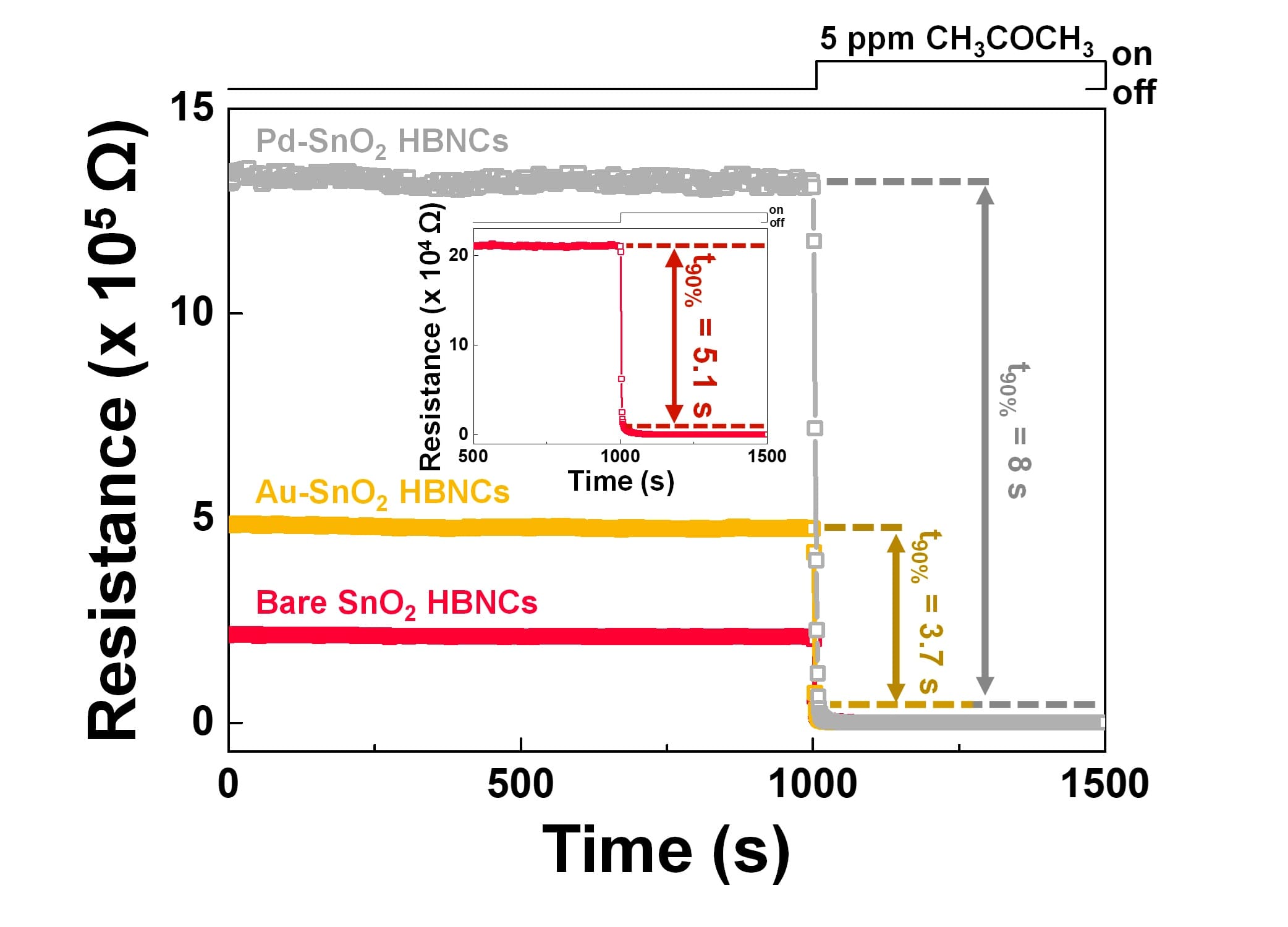


**Fig. S12**. Magnified resistance curves and response times of bare, Au-, and Pd-SnO_2_ HBNCs to 5 ppm CH_3_COCH_3_.


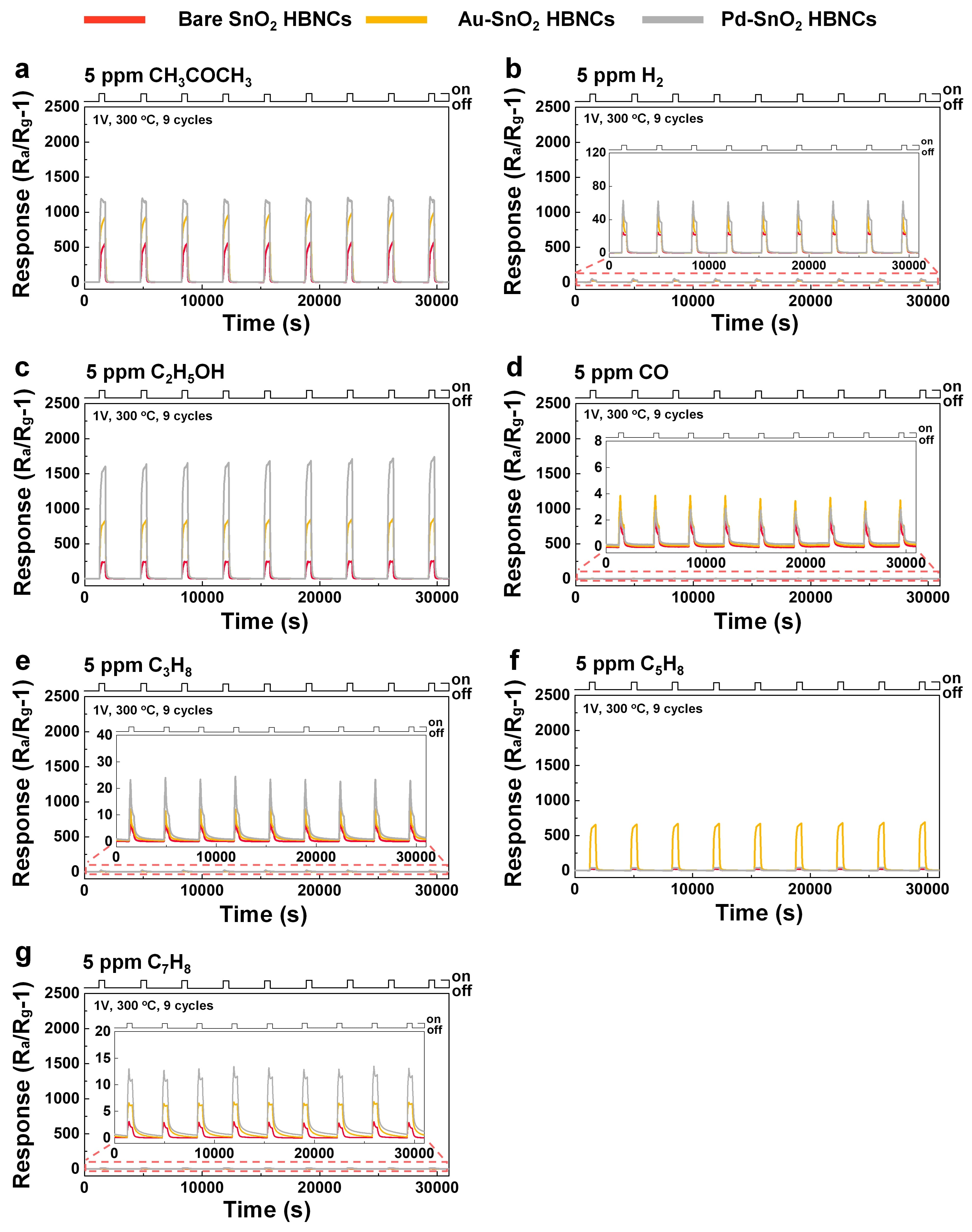


**Fig. S13.** Gas responses to seven different gases in dry condition. Cyclic response of bare, Au-, and Pd-SnO_2_ HBNCs to 5 ppm of **(a)** CH_3_COCH_3_, **(b)** H_2_, **(c)** C_2_H_5_OH, **(d)** CO, **(e)** C_3_H_8_, **(f)** C_5_H_8_, and **(g)** C_7_H_8_.


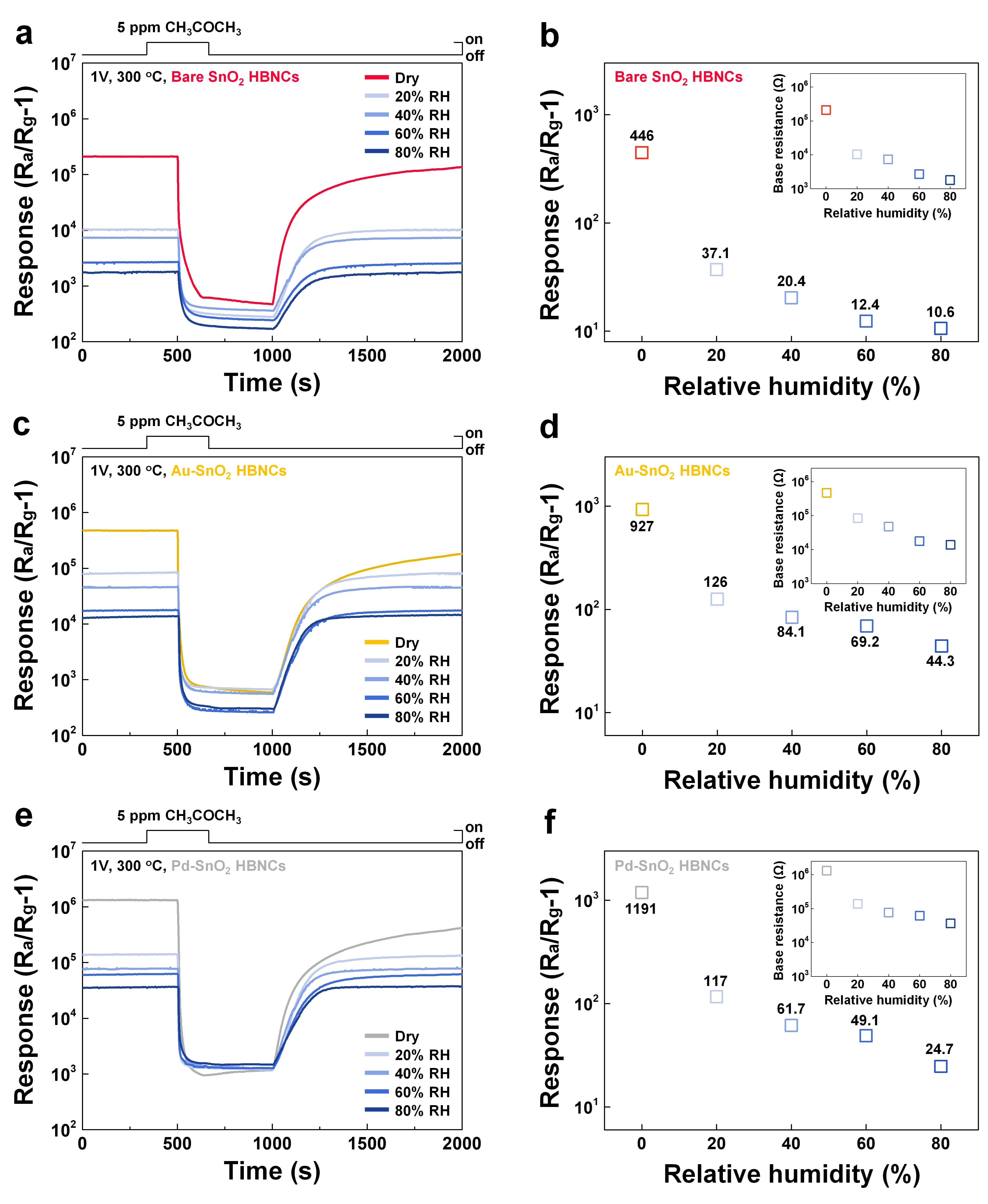


**Fig. S14.** Resistance curves and response of **(a, b)** bare SnO_2_ HBNCs, **(c, d)** Au-SnO_2_ HBNCs, and **(e, f)** Pd-SnO_2_ HBNCs to 5 ppm CH_3_COCH_3_ at 300 ^o^C as a function of RH: 0, 20, 40, 60, and 80%. The insets show the intrinsic resistance of bare, Au-, and Pd-SnO_2_ HBNCs under varying RH.


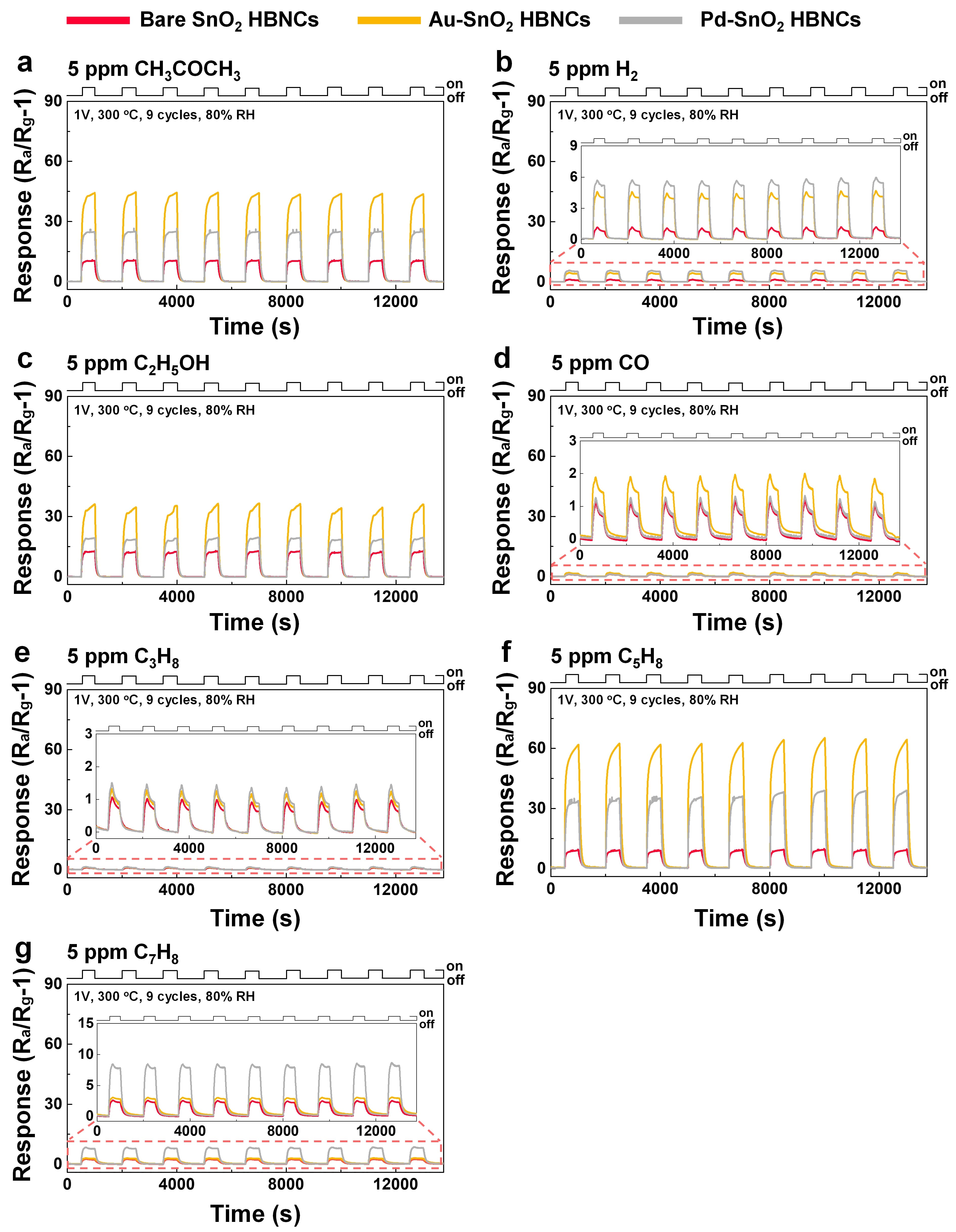
**Fig. S15.** Gas responses to seven different gases in 80% RH condition. Cyclic response of bare, Au-, and Pd-SnO_2_ HBNCs to 5 ppm of **(a)** CH_3_COCH_3_, **(b)** H_2_, **(c)** C_2_H_5_OH, **(d)** CO, **(e)** C_3_H_8_, **(f)** C_5_H_8_, and **(g)** C_7_H_8_.


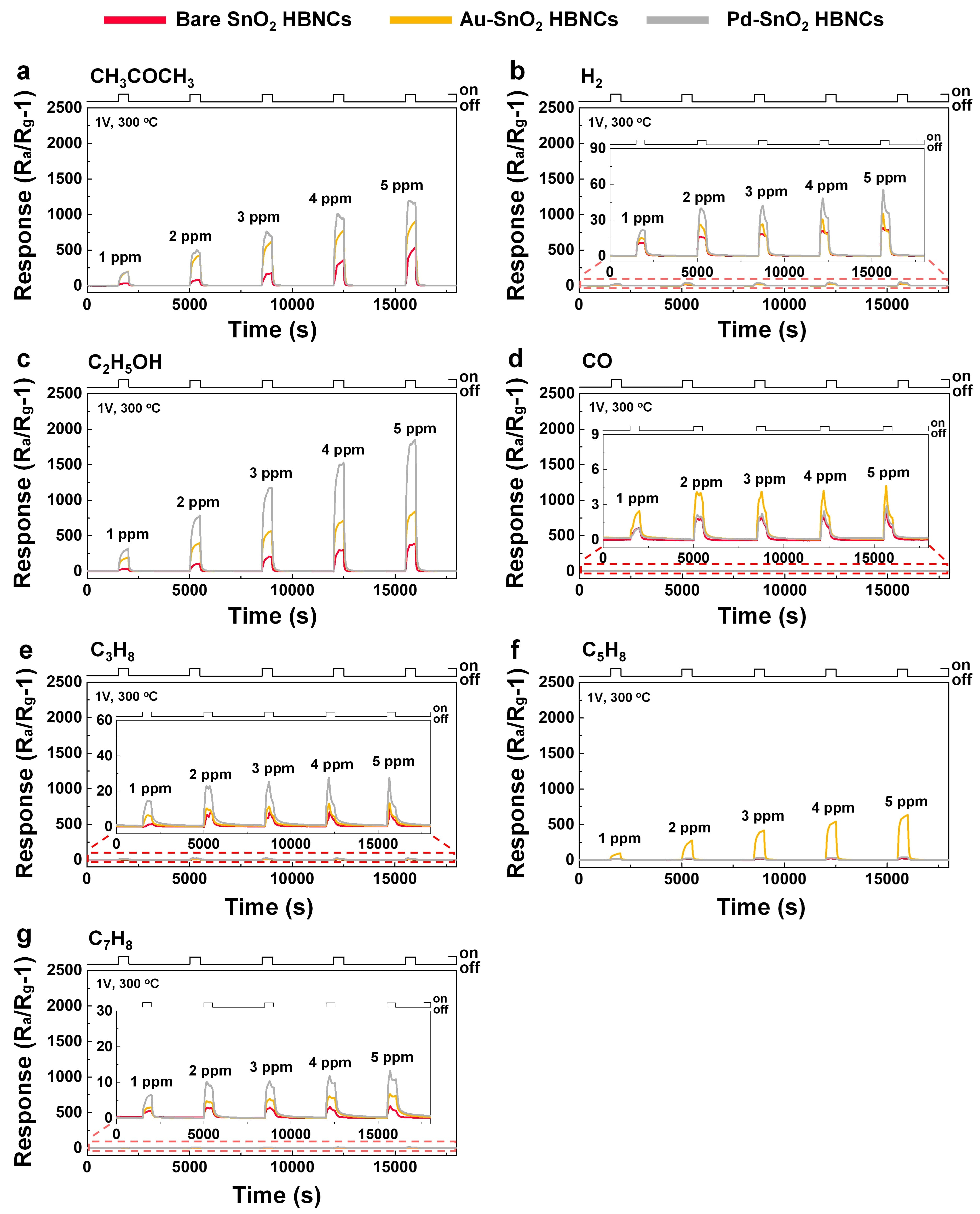
**Fig. S16**. Gas responses to seven different gases with controlled concentrations of 1-5 ppm. Response curves of bare, Au-, and Pd-SnO_2_ HBNCs to **(a)** CH_3_COCH_3_, **(b)** H_2_, **(c)** C_2_H_5_OH, **(d)** CO, **(e)** C_3_H_8_, **(f)** C_5_H_8_, and **(g)** C_7_H_8_.


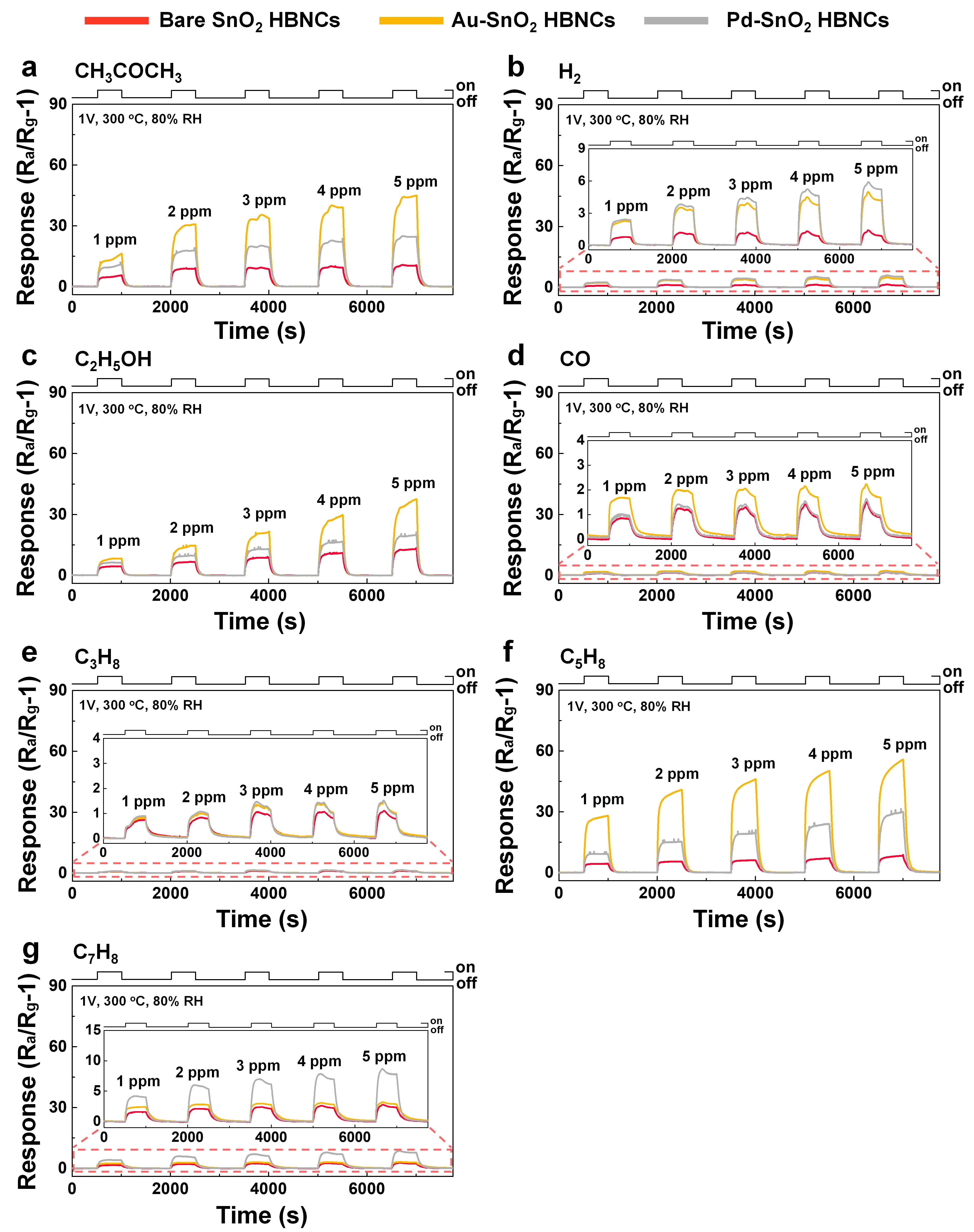
**Fig. S17.** Gas responses to seven different gases with controlled concentrations of 1-5 ppm under 80% RH. Response curves of bare, Au-, and Pd-SnO_2_ HBNCs to **(a)** CH_3_COCH_3_, **(b)** H_2_, **(c)** C_2_H_5_OH, **(d)** CO, **(e)** C_3_H_8_, **(f)** C_5_H_8_, and **(g)** C_7_H_8_.


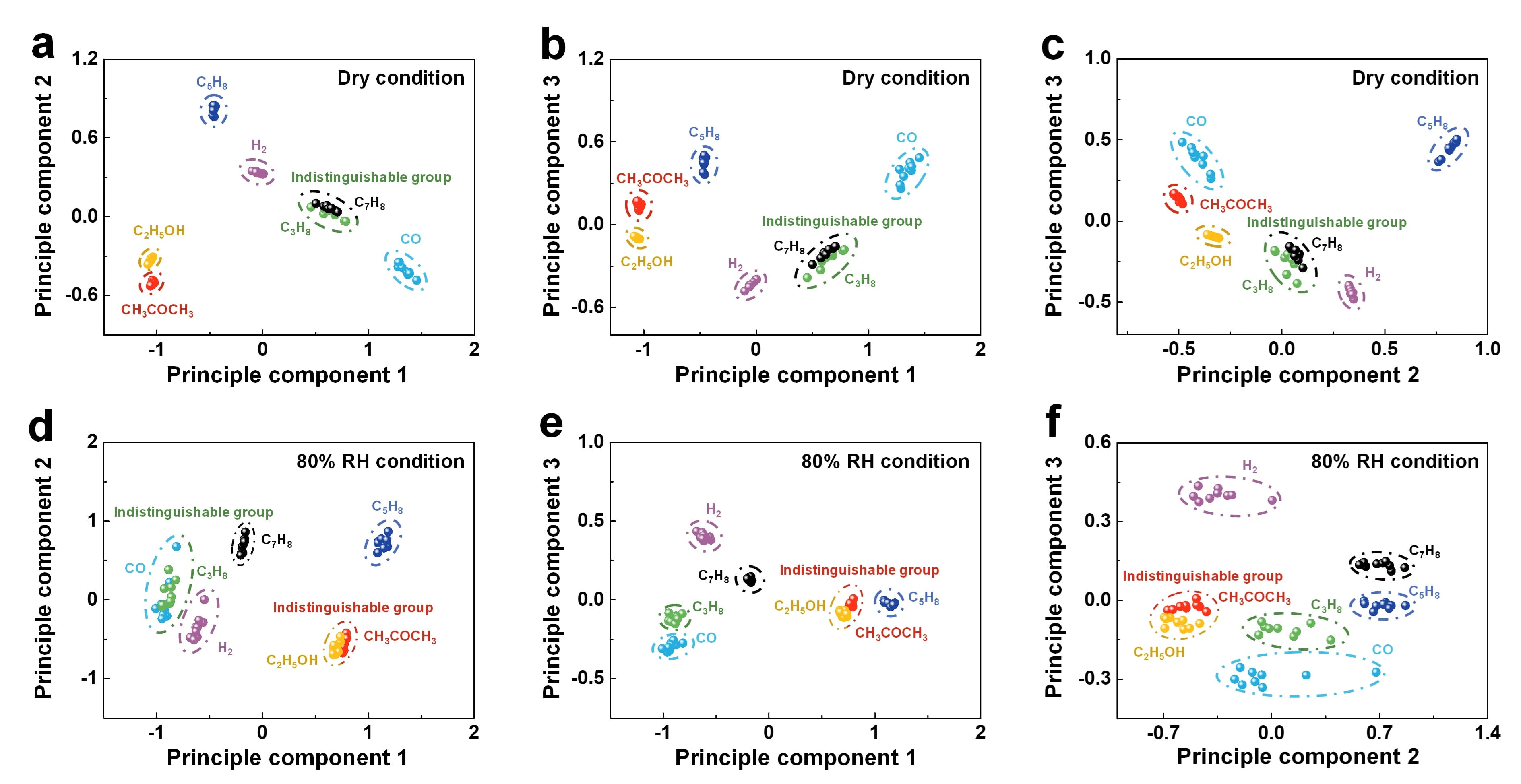


**Fig. S18**. 2D PCA results based on the response, response time, and recovery time from nine repeated measurements for seven gases at 5 ppm and 300 ^o^C for three samples under **(a-c)** dry and **(d-f)** 80% RH conditions.


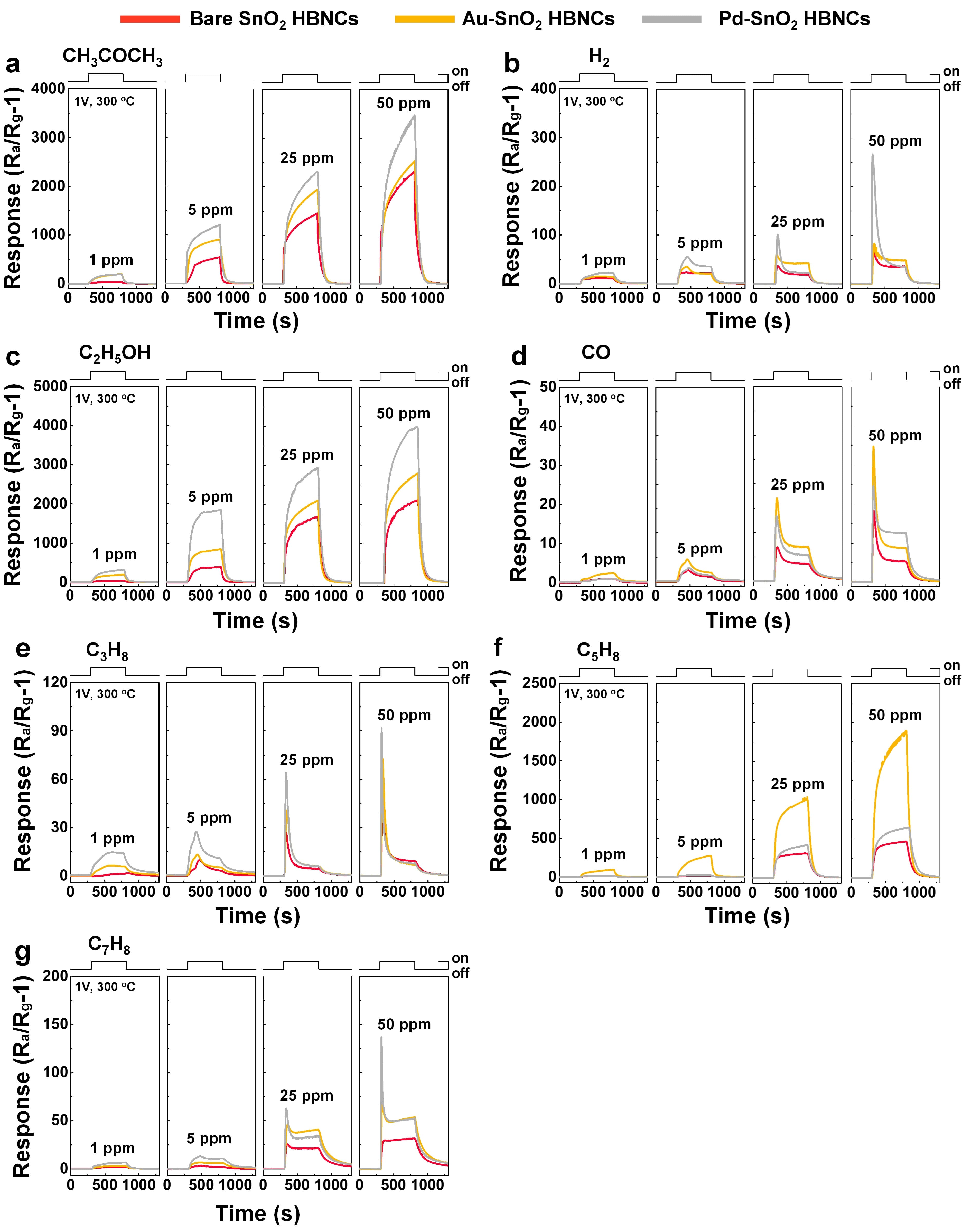


**Fig. S19**. Response curves to 1, 5, 25, and 50 ppm of seven gases for bare, Au-, and Pd-SnO_2_ HBNCs: **(a)** CH_3_COCH_3_, **(b)** H_2_, **(c)** C_2_H_5_OH, **(d)** CO, **(e)** C_3_H_8_, **(f)** C_5_H_8_, and **(g)** C_7_H_8_.


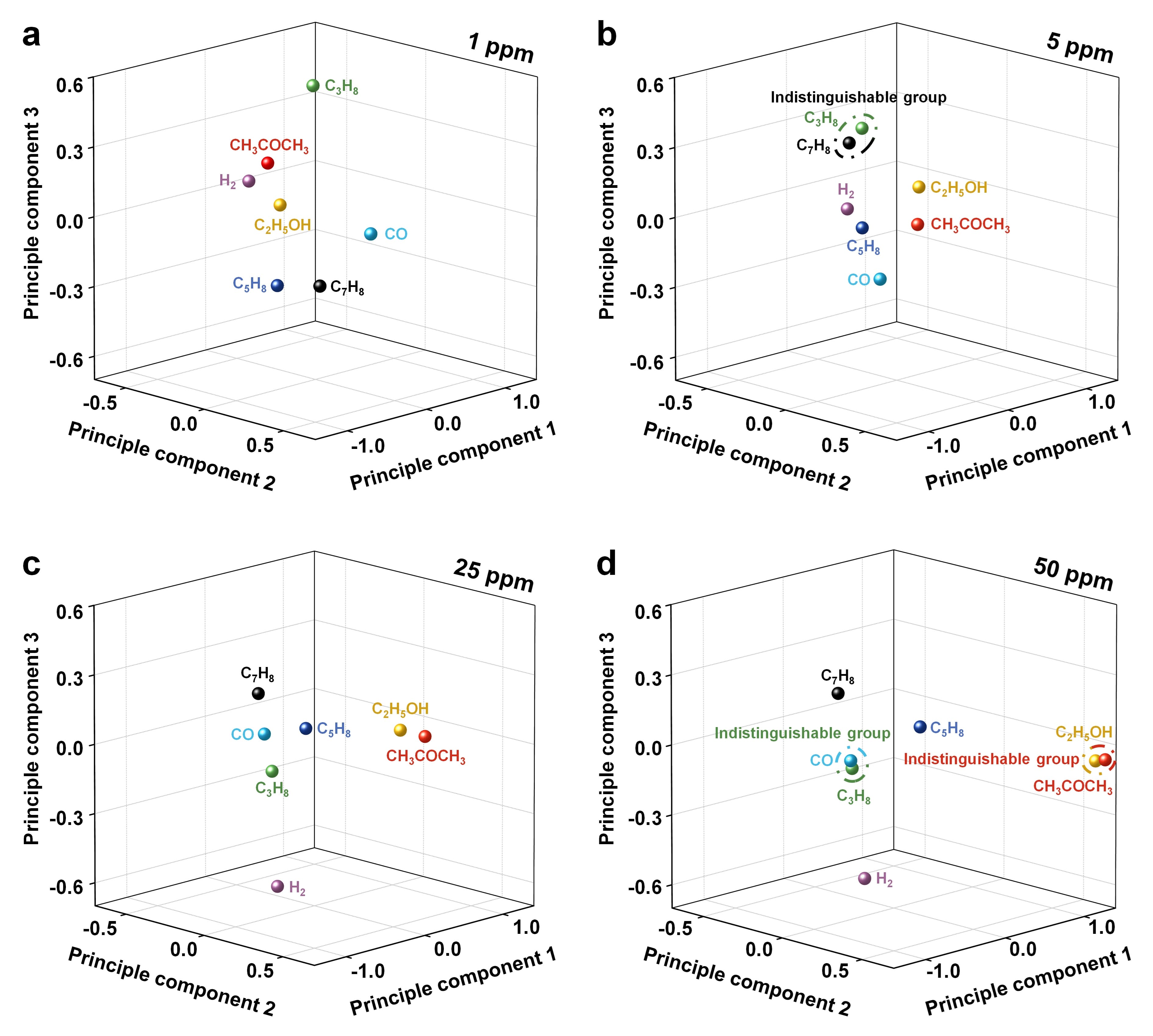


**Fig. S20.** 3D PCA results based on the response, response time, and recovery time for seven gases at **(a)** 1 ppm, **(b)** 5 ppm, **(c)** 25 ppm, and **(d)** 50 ppm, measured at 300 ^o^C for three samples.


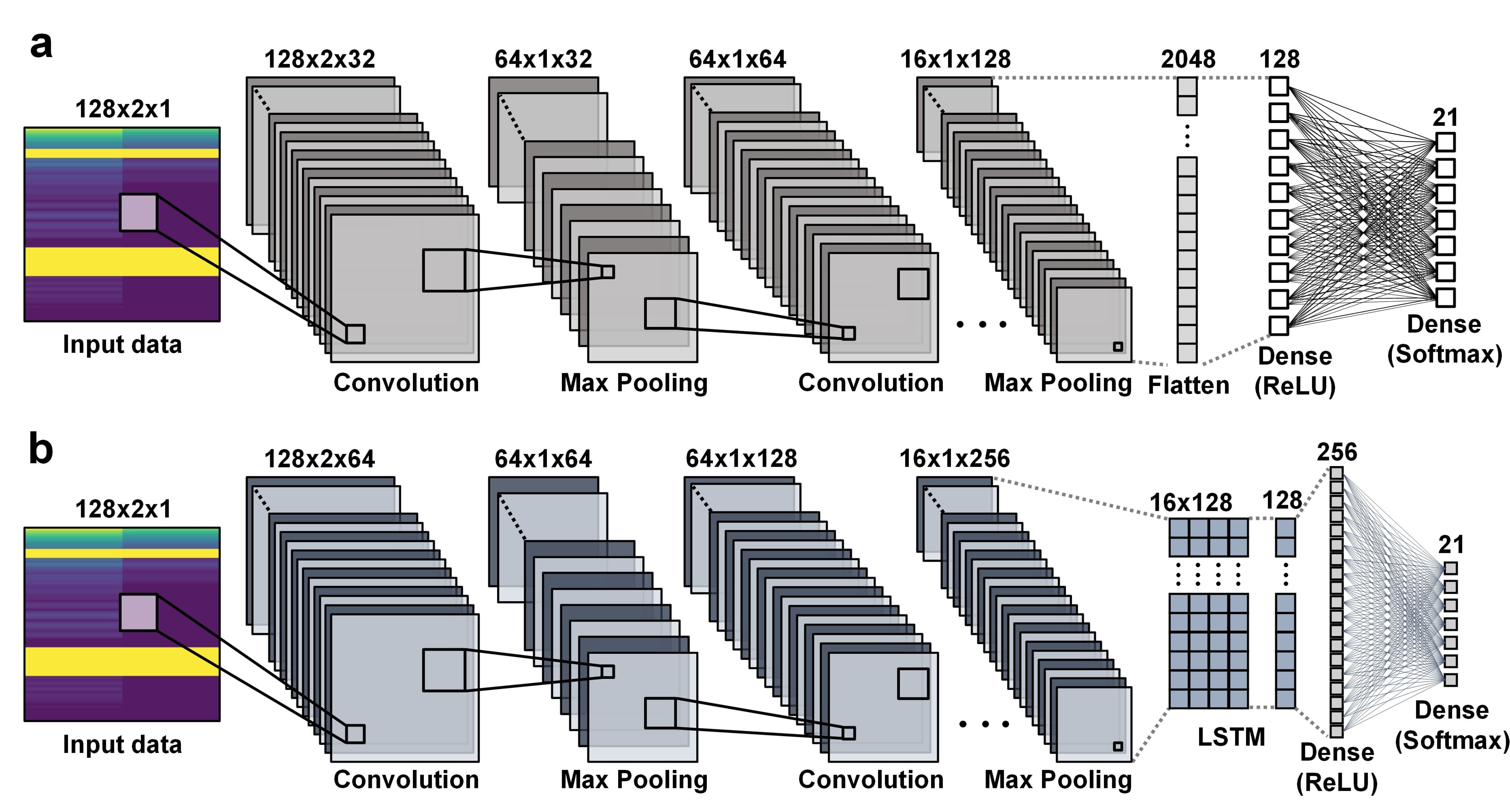


**Fig. S21.** Architectures of **(a)** the CNN model and **(b)** the CNN-LSTM model.


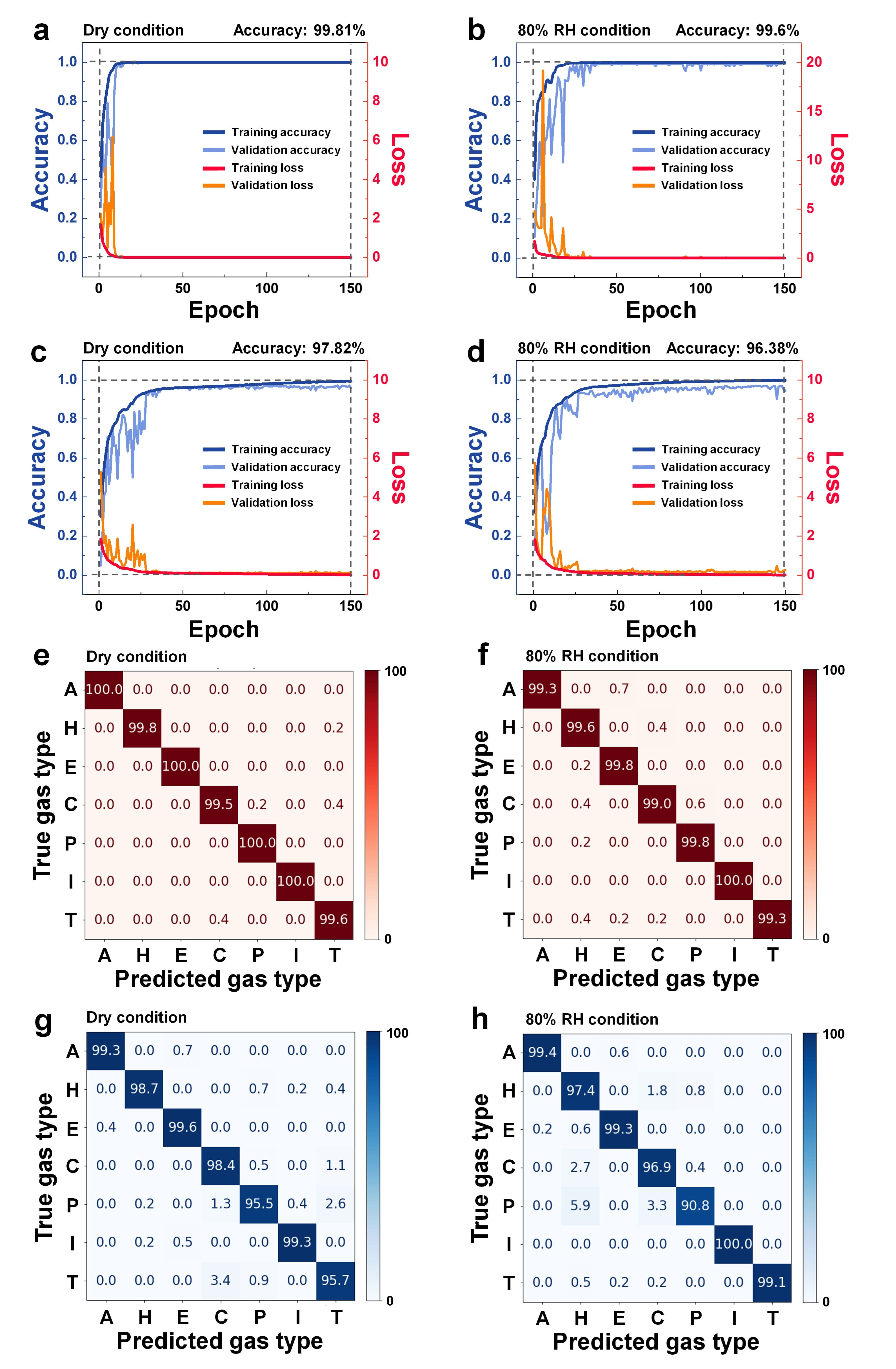


**Fig. S22.** Classification performances of three learning models. Accuracy and loss graphs for the CNN model under **(a)** dry and **(b)** 80% RH conditions, and for the CNN-LSTM model under **(c)** dry and **(d)** 80% RH conditions. Confusion matrices for the CNN model under **(e)** dry and **(f)** 80% RH conditions, and for the CNN-LSTM model under **(g)** dry and **(h)** 80% RH conditions (A: acetone, H: hydrogen, E: ethanol, C: carbon monoxide, P: propane, I: isoprene, and T: toluene).

**Supporting Tables**

| **Model** | **Model Size(MB)** | **Inference Time(ms/sample)** | **FLOPs (MFLOPs)** |
| --- | --- | --- | --- |
| CNN | 4.16 | 59.53 | 7.83 |
| CNN-LSTM | 8.52 | 84.51 | 28.83 |
| RESNET | 6.70 | 73.70 | 27.57 |

**Table S1.** Comparison of model storage, inference time, and computational cost.

| **Target gas** | | **CH_3_COCH_3_** | **H_2_** | **C_2_H_5_OH** | **CO** | **C_3_H_8_** | **C_5_H_8_** | **C_7_H_8_** |
| --- | --- | --- | --- | --- | --- | --- | --- | --- |
| Bare SnO_2_ HBNCs | rms | 0.000247 | 0.000402 | 0.000872 | 0.000904 | 0.001309 | 0.002495 | 0.000928 |
|  | slope | 129.4 | 3.05 | 90.63 | 0.31 | 1.71 | 2.43 | 0.29 |
| Au-SnO_2_ HBNCs | rms | 0.001571 | 0.002643 | 0.001381 | 0.000427 | 0.000873 | 0.001606 | 0.000578 |
|  | slope | 176.6 | 5.01 | 160.09 | 0.44 | 1.59 | 135.07 | 0.89 |
| Pd-SnO_2_ HBNCs | rms | 0.003452 | 0.002841 | 0.002309 | 0.002209 | 0.000659 | 0.000975 | 0.003377 |
|  | slope | 251.2 | 7.99 | 984.09 | 0.41 | 3.01 | 5.84 | 1.51 |

**Table S2.** Summary of rms and slope values for seven gases across three samples.

| **Target gas** | | **CH_3_COCH_3_** | **H_2_** | **C_2_H_5_OH** | **CO** | **C_3_H_8_** | **C_5_H_8_** | **C_7_H_8_** |
| --- | --- | --- | --- | --- | --- | --- | --- | --- |
| Bare SnO_2_ HBNCs | rms | 0.000148 | 0.000183 | 0.000995 | 0.000641 | 0.001068 | 0.0006766 | 0.0005363 |
|  | slope | 1.1478 | 0.1415 | 2.1875 | 0.1445 | 0.0964 | 0.9264 | 0.2894 |
| Au-SnO_2_ HBNCs | rms | 0.000188 | 0.000123 | 0.000735 | 0.000438 | 0.007336 | 0.0007825 | 0.000145 |
|  | slope | 6.6753 | 0.6395 | 7.3431 | 0.1231 | 0.1679 | 6.4705 | 0.1916 |
| Pd-SnO_2_ HBNCs | rms | 0.00014 | 0.002226 | 0.000355 | 0.000302 | 0.007754 | 0.000248 | 0.000574 |
|  | slope | 3.2931 | 0.8322 | 3.4276 | 0.8958 | 0.1605 | 5.0418 | 1.0865 |

**Table S3.** Summary of rms and slope values for seven gases across three samples under 80% RH conditions.

| **Layer** | **Type** | **Filters**  **/Units** | **Kernel Size** | **Pool Size** | **Strides** | **Activation** | **Output Shape** |
| --- | --- | --- | --- | --- | --- | --- | --- |
| Input | Input Layer | - | - | - | - | - | (128,2,1) |
| Conv2D_1 | Convolutional | 32 | (3,3) | - | (1,1) | ReLU | (128,2,32) |
| BatchNormalization_1 | Batch Normalization | - | - | - | - | - | (128,2,32) |
| MaxPooling2D_1 | Max Pooling | - | - | (2,2) | (2,2) | - | (64,1,32) |
| Conv2D_2 | Convolutional | 64 | (3,3) | - | (1,1) | ReLU | (64,1,64) |
| BatchNormalization_2 | Batch Normalization | - | - | - | - | - | (64,1,64) |
| MaxPooling2D_2 | Max Pooling | - | - | (2,2) | (2,2) | - | (32,1,64) |
| Conv2D_3 | Convolutional | 128 | (3,3) | - | (1,1) | ReLU | (32,1,128) |
| BatchNormalization_3 | Batch Normalization | - | - | - | - | - | (32,1,128) |
| MaxPooling2D_3 | Max Pooling | - | - | (2,2) | (2,2) | - | (16,1,128) |
| Flatten | Flatten | - | - | - | - | - | (128) |
| Dropout | Dropout | - | - | - | - | - | (2048) |
| Dense_1 | Fully Connected | 128 | - | - | - | ReLU | (128) |
| Dense_2 | Fully Connected | 21 |  |  |  | Softmax | (21) |

**Table S4.** Detailed architecture of the CNN model.

| **Layer** | **Type** | **Filters**  **/Units** | **Kernel Size** | **Strides** | **Activation** | **Output Shape** |
| --- | --- | --- | --- | --- | --- | --- |
| Input | Input Layer | - | - | - | - | (128,2,1) |
| Conv2D_1 | Convolutional | 64 | (3,3) | (1,1) | ReLU | (128,2,64) |
| BatchNormalization_1 | Batch Normalization | - | - | - | - | (128,2,64) |
| MaxPooling2D_1 | Max Pooling | - | (2,2) | (2,2) | - | (64,1,64) |
| Conv2D_2 | Convolutional | 128 | (3,3) | (1,1) | ReLU | (64,1,128) |
| BatchNormalization_2 | Batch Normalization | - | - | - | - | (64,1,128) |
| MaxPooling2D_2 | Max Pooling | - | (2,2) | (2,2) | - | (32,1,128) |
| Conv2D_3 | Convolutional | 256 | (3,3) | (1,1) | ReLU | (32,1,256) |
| BatchNormalization_3 | Batch Normalization | - | - | - | - | (32,1,256) |
| MaxPooling2D_3 | Max Pooling | - | (2,2) | (2,2) | - | (16,1,256) |
| Reshape | Reshape | - | - | - | - | (16,256) |
| LSTM_1 | LSTM (Return Sequences) | 128 | - | - | - | (16,128) |
| LSTM_2 | LSTM | 128 |  |  | - | (128) |
| Dense_1 | Fully Connected | 256 |  |  | ReLU | (256) |
| Dropout | Dropout | - | - | - | - | (256) |
| Dense_2 | Fully Connected | 21 | - | - | Softmax | (21) |

**Table S5.** Detailed architecture of the CNN-LSTM model.

| **Layer** | **Type** | **Filters/Units** | **Kernel Size** | **Strides** | **Activation** | **Output Shape** |
| --- | --- | --- | --- | --- | --- | --- |
| Input | Input Layer | - | - | - | - | (128,2,1) |
| Conv2D_1 | Convolutional | 32 | (3,3) | (1,1) | ReLU | (128,2,32) |
| BatchNormalization_1 | Batch Normalization | - | - | - | - | (128,2,32) |
| MaxPooling2D_1 | Max Pooling | - | (2,1) | (2,1) | - | (64,2,32) |
| Residual Block 1 | Convolutional Block | 32 | (3,3) | (1,1) | ReLU | (64,2,32) |
| Residual Block 2 | Convolutional Block | 64 | (3,3) | (2,1) | ReLU | (32,2,64) |
| Residual Block 3 | Convolutional Block | 128 | (3,3) | (2,1) | ReLU | (16,2,128) |
| MaxPooling2D_2 | Max Pooling | - | (2,1) | (2,1) | - | (8,2,128) |
| Flatten | Flatten | - | - | - | - | (2048) |
| Dense_1 | Fully Connected | 128 | - | - | ReLU | (128) |
| Dropout | Dropout | - | - | - | - | (128) |
| Dense_2 | Fully Connected | 21 |  |  | Softmax | (21) |

**Table S6.** Detailed architecture of the ResNet model.
